# Facile and Green Synthesis of Saturated Cyclic Amines

**DOI:** 10.3390/molecules22101691

**Published:** 2017-10-12

**Authors:** Arruje Hameed, Sadia Javed, Razia Noreen, Tayyaba Huma, Sarosh Iqbal, Huma Umbreen, Tahsin Gulzar, Tahir Farooq

**Affiliations:** 1 Department of Biochemistry, Government College University, Faisalabad 38900, Pakistan; arrujetahirfsd@gmail.com (A.H.); diyajav1@yahoo.com (S.J.); itsrazia@yahoo.com (R.N.); 2 Department of Bioinformatics and Biotechnology, Government College University, Faisalabad 38900, Pakistan; tayyabahuma@gcuf.edu.pk; 3 Department of Applied Chemistry, Government College University, Faisalabad 38900, Pakistan; sarosh.iqbal@yahoo.com (S.I.); tahsingulzar1@yahoo.com (T.G.); 4 Department of Home Economics, Government College Women University, Faisalabad 38900, Pakistan; huma.umbreen@yahoo.com

**Keywords:** cyclic amines, green synthesis, one-pot reactions, microwave assisted, solvent free

## Abstract

Single-nitrogen containing saturated cyclic amines are an important part of both natural and synthetic bioactive compounds. A number of methodologies have been developed for the synthesis of aziridines, azetidines, pyrrolidines, piperidines, azepanes and azocanes. This review highlights some facile and green synthetic routes for the synthesis of unsubstituted, multisubstituted and highly functionalized saturated cyclic amines including one-pot, microwave assisted, metal-free, solvent-free and in aqueous media.

## 1. Introduction

**Figure 1:**
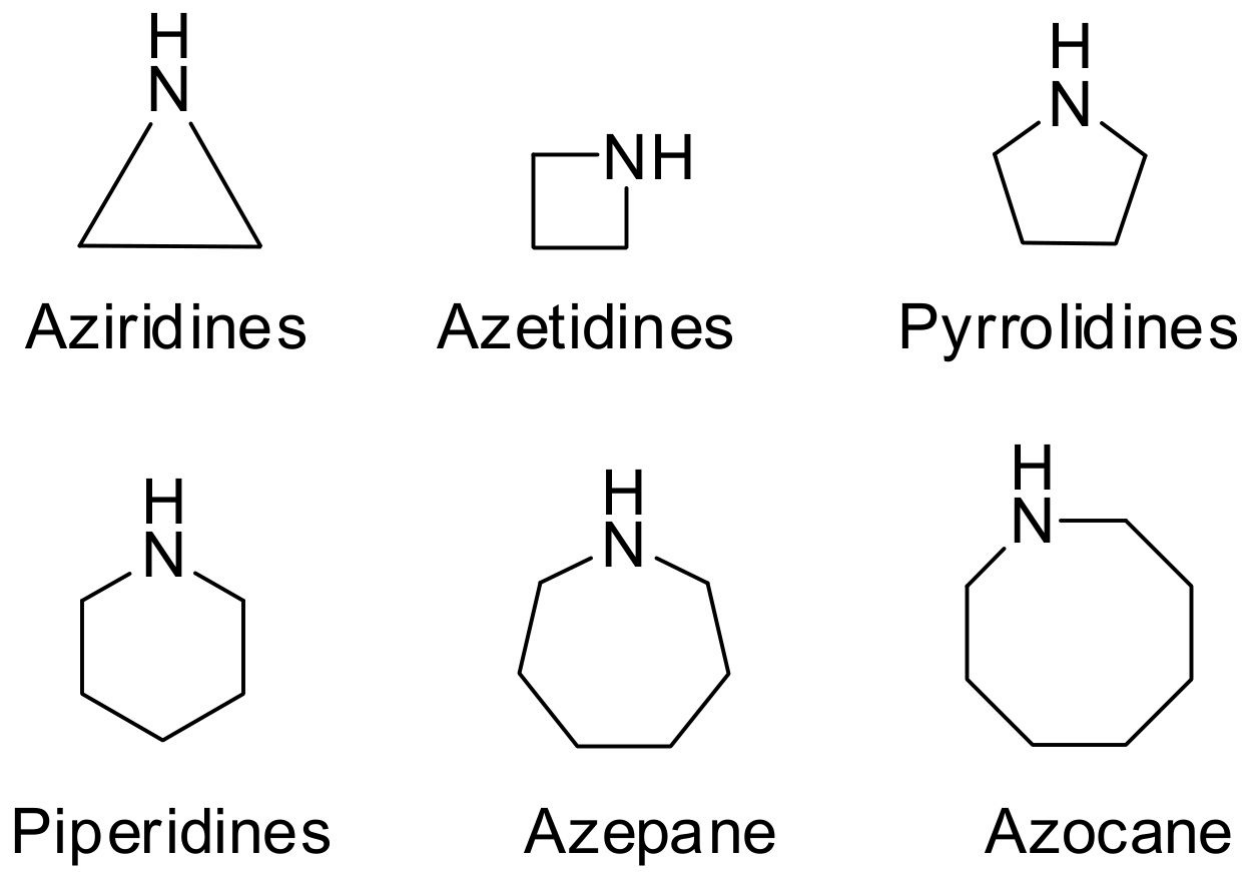
Saturated cyclic amines containing one-nitrogen atom.

**Figure 2:**
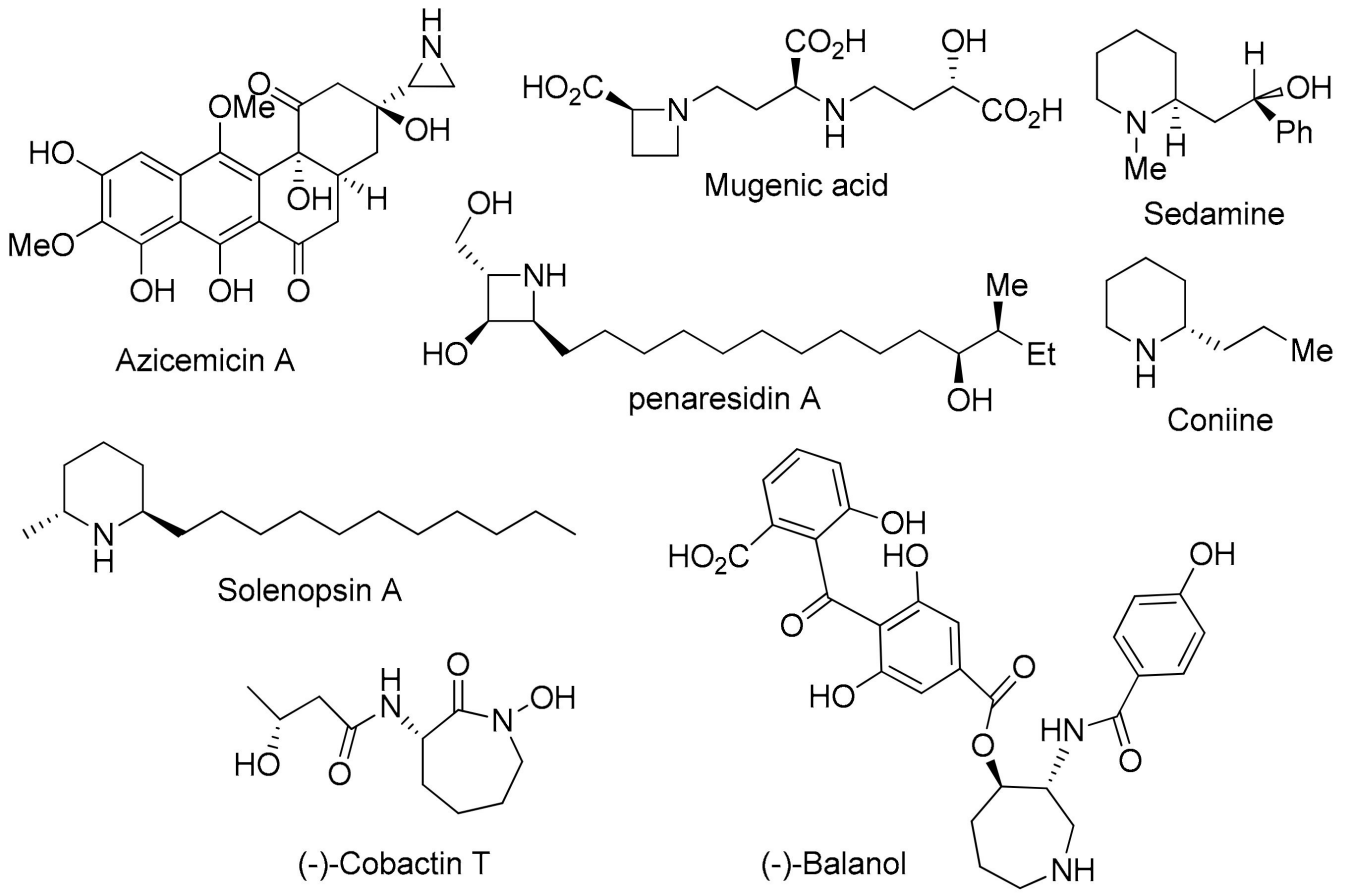
Naturally compounds containing saturated cyclic amines.

**Figure 3:**
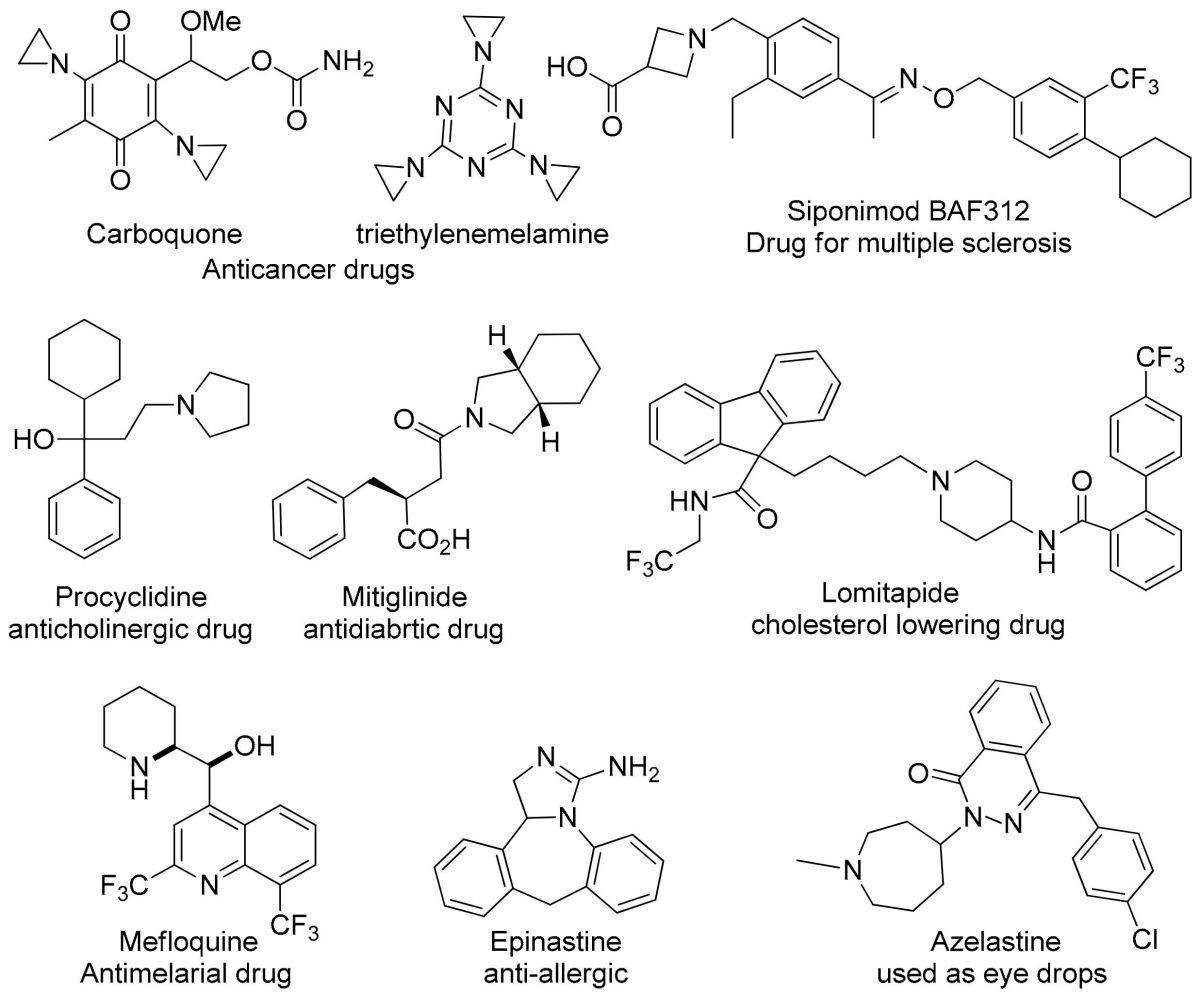
Clinical drugs containing saturated cyclic amine.

Saturated cyclic amines are prominent bioactive motifs in many medicinal compounds and natural products (Figure 1 and Figure 2). They are also attractive building blocks employed to construct other molecules of medicinal or biological interest [1]. A large number of such building blocks have also been used in polymers [2], copolymers [3], dyestuffs [4], reaction media [5], bases and chiral ligands, etc. [6,7,8]. Further, a large number of pharmacologically active natural and synthetic cyclic amines are in regular clinical use (Figure 3) [9]. They have been utilized as antibiotics, anticancer, analgesics, antidepressants, anti-HIV, anti-HCV agents, etc. [10,11]. A variety of cyclic amine-containing compounds also find applications as insecticides, pesticides, rodenticides and herbicides [12,13,14,15,16].

A broad scope of applications of the aforementioned saturated cyclic amines has revolutionized the synthetic methodologies used to access such compounds. A number of efficient synthetic methods have been developed over the past decades [17,18,19]. In recent years, quite a many environmentally benign methods have also been developed for the synthesis of saturated cyclic amines. This review aims to highlight the green synthetic methodologies including one-pot, multi-component, solvent-free, microwave-assisted and synthesis in aqueous media for single-nitrogen containing saturated cyclic amines.

## 2. Green Methods for Synthesis of Aziridines

**Scheme 1:**
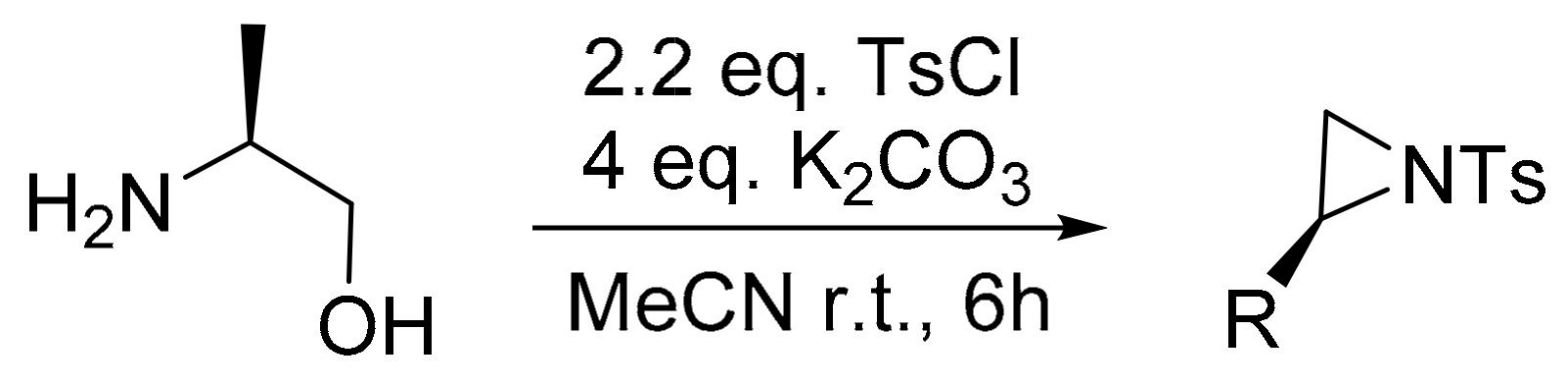
One-pot synthesis of *N*-tosylaziridine.

**Scheme 2:**
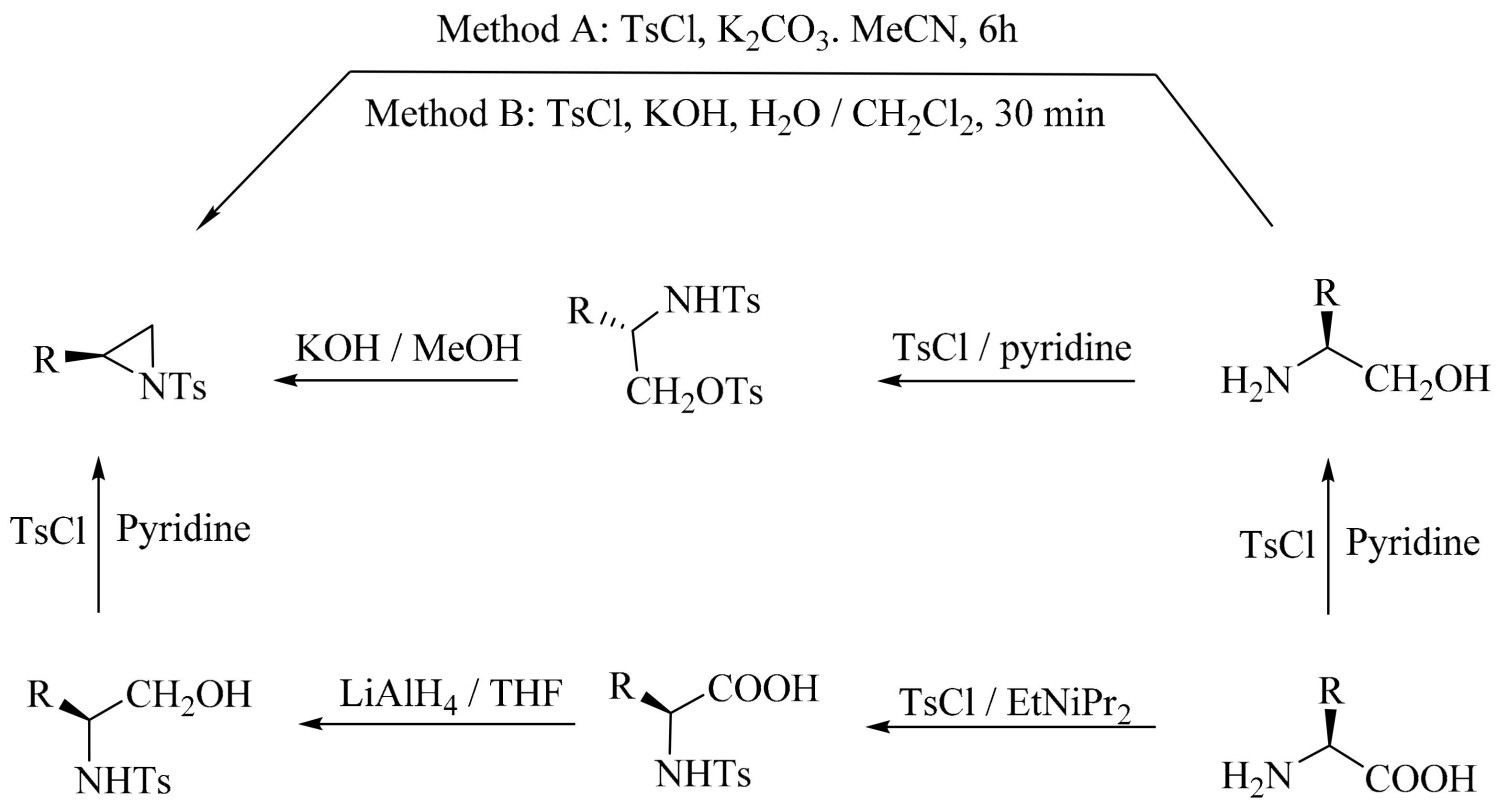
Transformation of aminoalcohols to *N*-tosylaziridines.

Bieber et al. presented a one-pot method for the synthesis of *N*-tosylaziridines from 2-amino-alcohols [20]. This one-pot transformation involves the tosylation and cyclization steps (Scheme 1). It was observed that highly substituted amino alcohols produce the corresponding aziridines in high yields in acetonitrile in the presence of K_2_CO_3_. High yields of less hindered aziridines were obtained from the corresponding alcohols when the reactions were performed in H_2_O/DCM using KOH (Scheme 2).

**Scheme 3:**
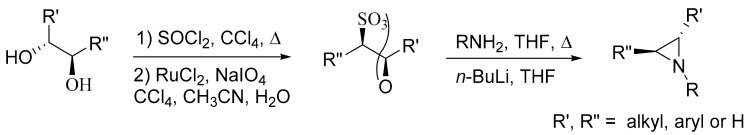
One-pot synthesis of aziridines from cyclic sulfates.

Lohray and his research group used 1,2-cyclic sulfates for the one-pot synthesis of aminoalcohols and homochiral *N*-substituted aziridines (Scheme 3). Cyclic sulfates react with primary amines to furnish β-aminosulfates which are converted to aziridines under basic conditions [21].

**Scheme 4:**
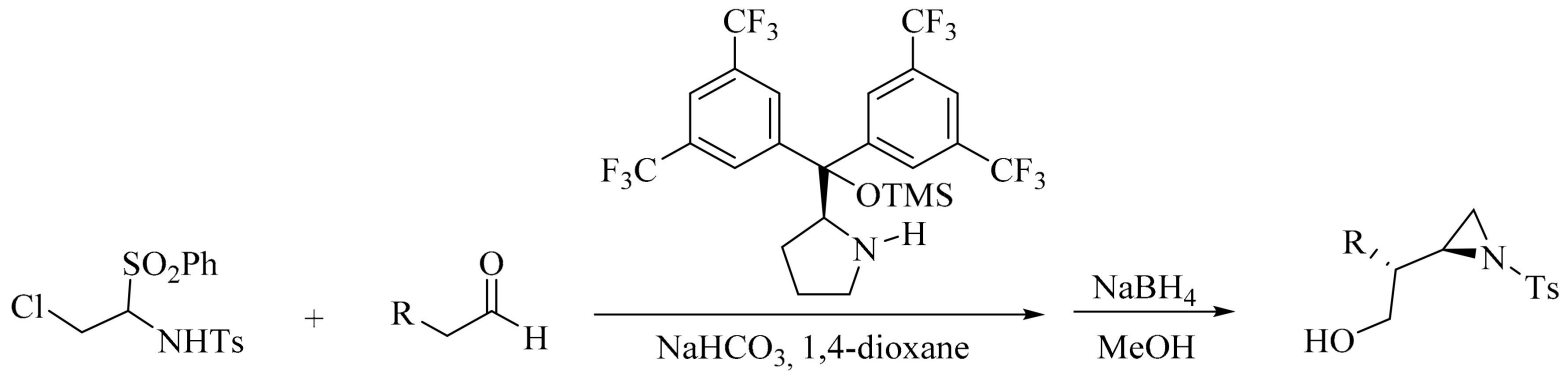
Synthesis of chiral *N*-tosylated aziridines.

Hayashi and his co-workers adopted a one-pot procedure to produce chiral aziridines with excellent enantio- and diastereoselectivities (Scheme 4). The sequential reactions include the desulfonylative generation of the *N*-tosylated imine from chloroacetaldehyde, an asymmetric Mannich reaction mediated by diarylprolinol silyl ether, reduction and finally aziridine formation [22].

**Scheme 5:**
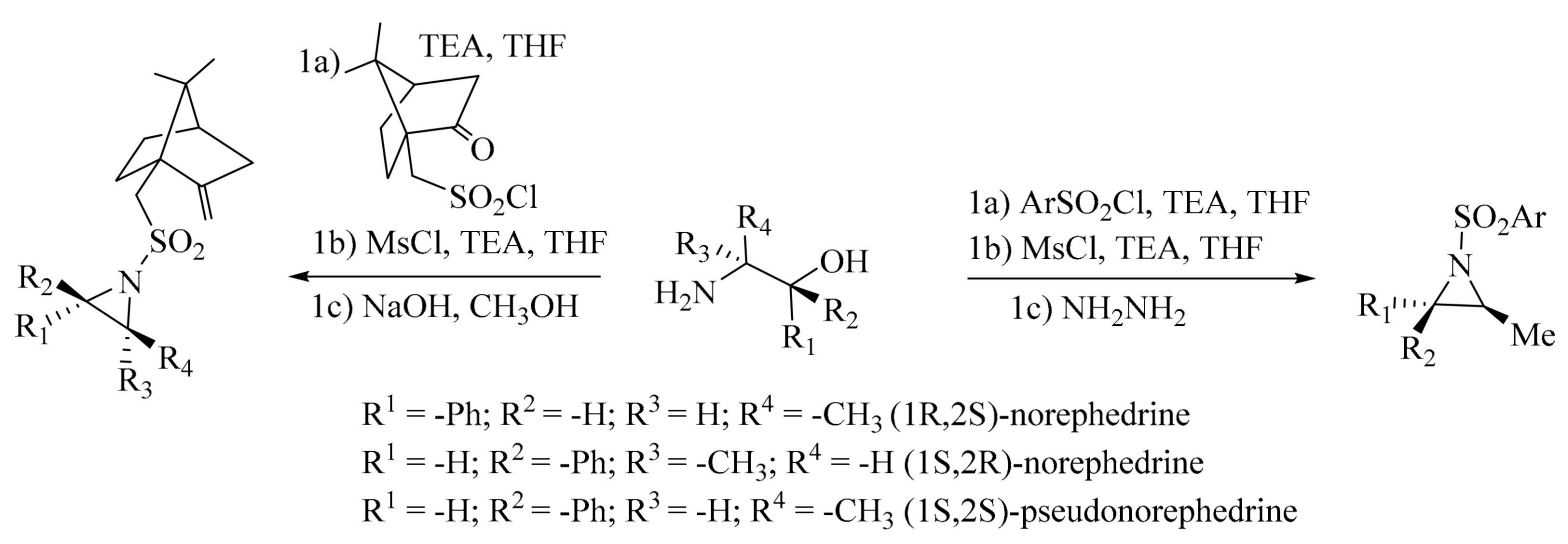
Synthesis of *N*-sulfonylaziridines.

Using a one-pot synthesis strategy, a series of (1*R*,2*S*)- and (1*S*,2*R*)-norephedrine and (1*S*,2*S*)-pseudonorephedrine were converted into diastereomerically and enantiomerically enriched *N*-sulfonylaziridines (Scheme 5). The reaction involves the *N*-sulfonylation of the Ephedra alkaloid followed by *O*-sulfonylation with methanesulfonyl chloride [23]. The bis(sulfonyl) *Ephedra* derivatives afforded the corresponding *N*-sulfonylaziridines when they were treated with sodium hydroxide or hydrazine.

**Scheme 6:**
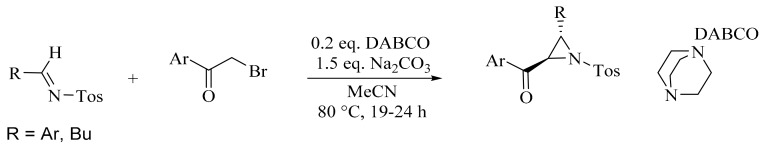
Synthesis of functionalized aziridines.

Yadav et al. described a one-pot process for the synthesis of functionalized aziridines by the reaction of imines with phenacyl bromide derivatives. This high yielding stereoselective reaction is catalyzed by the tertiary amine DABCO (Scheme 6) [24].

**Scheme 7:**
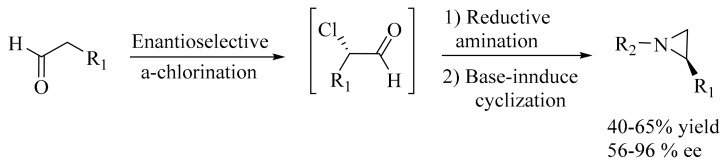
Synthesis of *N*-alkyl terminal aziridines.

**Scheme 8:**
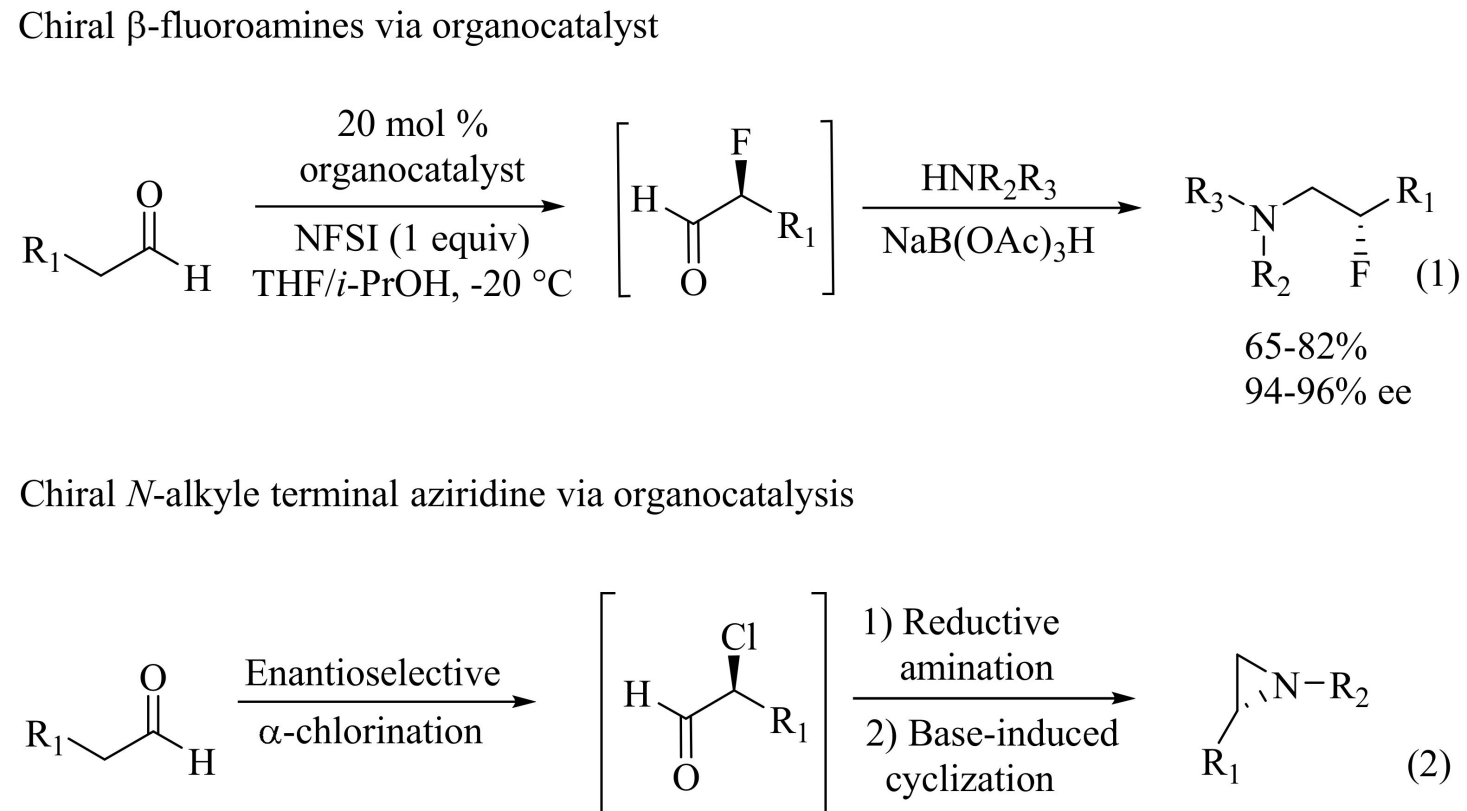
Organocatalytic approach to chiral *N*-alkyl terminal aziridines.

Olugbeminiyi and his co-workers presented a one-pot methodology that affords chiral *N*-alkyl terminal aziridines with more than 90% ee (Scheme 7). During this three step reaction, aldehydes initially undergo an enantioselective α-chlorination then reductive amination with primary amines and finally SN_2_ displacement results in the formation of the *N*-alkyl terminal aziridines (Scheme 8) [25].

**Scheme 9:**
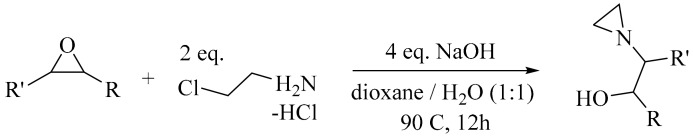
Synthesis of *N*-β-hydroxyethylaziridines.

A convenient method for the synthesis of *N*-β-hydroxyethylaziridines was presented by Kim and his co-workers. *N*-β-Hydroxyethylaziridines are known as important biological intermediates. They found that various epoxides undergo regioselective ring reactions with ethyleneimines. Under basic conditions, the ethyleneimine is generated in-situ from β-chloroethylamine in an aqueous environment (Scheme 9) [26].

**Scheme 10:**
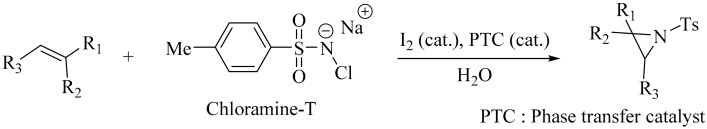
Synthesis of substituted aziridines from olefins.

Aziridines with a range of substituents were synthesized from the corresponding olefins in aqueous media. The iodine-catalyzed reaction requires quaternary ammonium salt in catalytic amounts to furnish good yield of highly pure product with simple work-up (Scheme 10) [27].

**Scheme 11:**
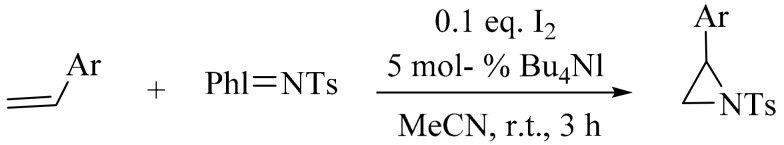
Synthesis of *N*-tosyl aziridines from styrene.

Kiyokawa and his research fellows presented that styrene derivatives do react with *N*-tosyliminophenyliodinane to furnish *N*-tosyl aziridines in good yields. This metal-free catalytic transformation is promoted by a combination of tetrabutylammonium iodide (TBAI) and I_2_ (Scheme 11).

During this reaction, both an extremely efficient catalyst TBAI_3_ and a real aziridination reagent called *N,N*-diiodotosylamide are produced in situ [28].

**Scheme 12:**
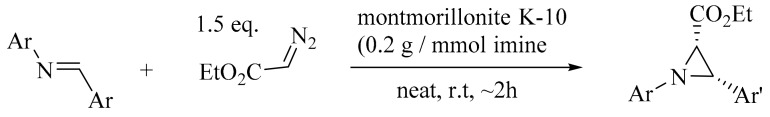
Synthesis of *cis*-aziridines from imines.

Imines react stereoselectively with ethyl diazoacetate in a montmorillonite K-10 solid acid catalyzed reaction to produce *cis*-aziridines in high yields (Scheme 12). This highly selective reaction accomplishes in short time under solvent-free conditions at room temperature furnishing the product in good yield [29].

## 3. Green Synthesis of Azetidines

**Scheme 13:**
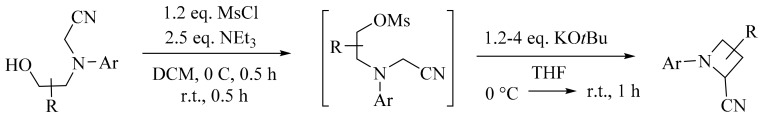
Synthesis of *N*-aryl-2-cyanoazetidines.

**Scheme 14:**
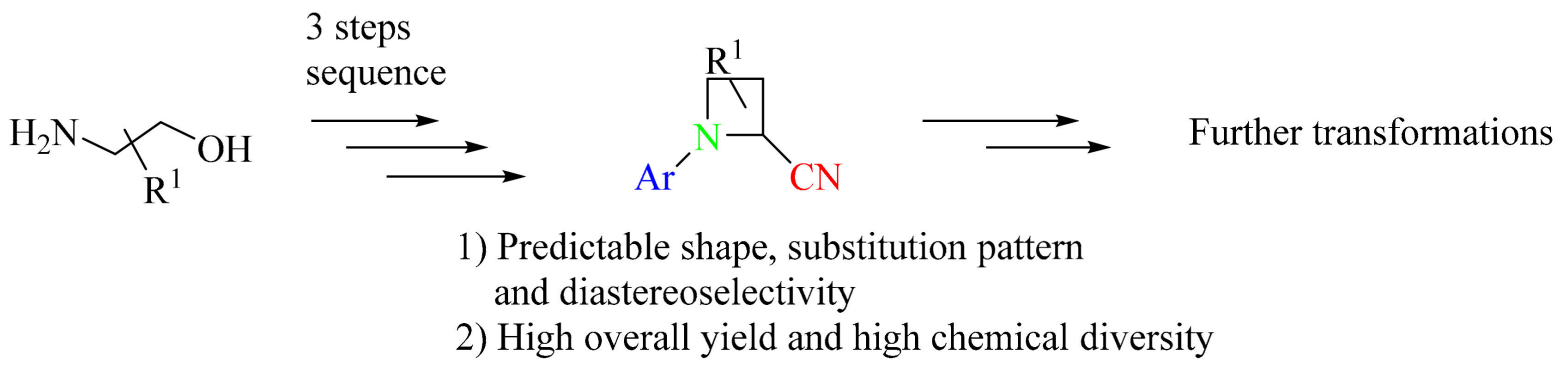
Sequential transformation of β-amino alcohols.

Quinodoze and his colleagues demonstrated an easily practicable method to access enantiomerically pure *N*-aryl-2-cyanoazetidines with a range of substituents in high yield. This three-step transformation of β-amino alcohol proceeds through Cu-catalyzed *N*-arylation, *N*-cyanomethylation of aniline and mesylation followed by base induced ring closure (Scheme 13 and Scheme 14). The azetidine product with desired substitution pattern and diastereoselectivity signifies the versatility of this highly efficient reaction [30].

**Scheme 15:**
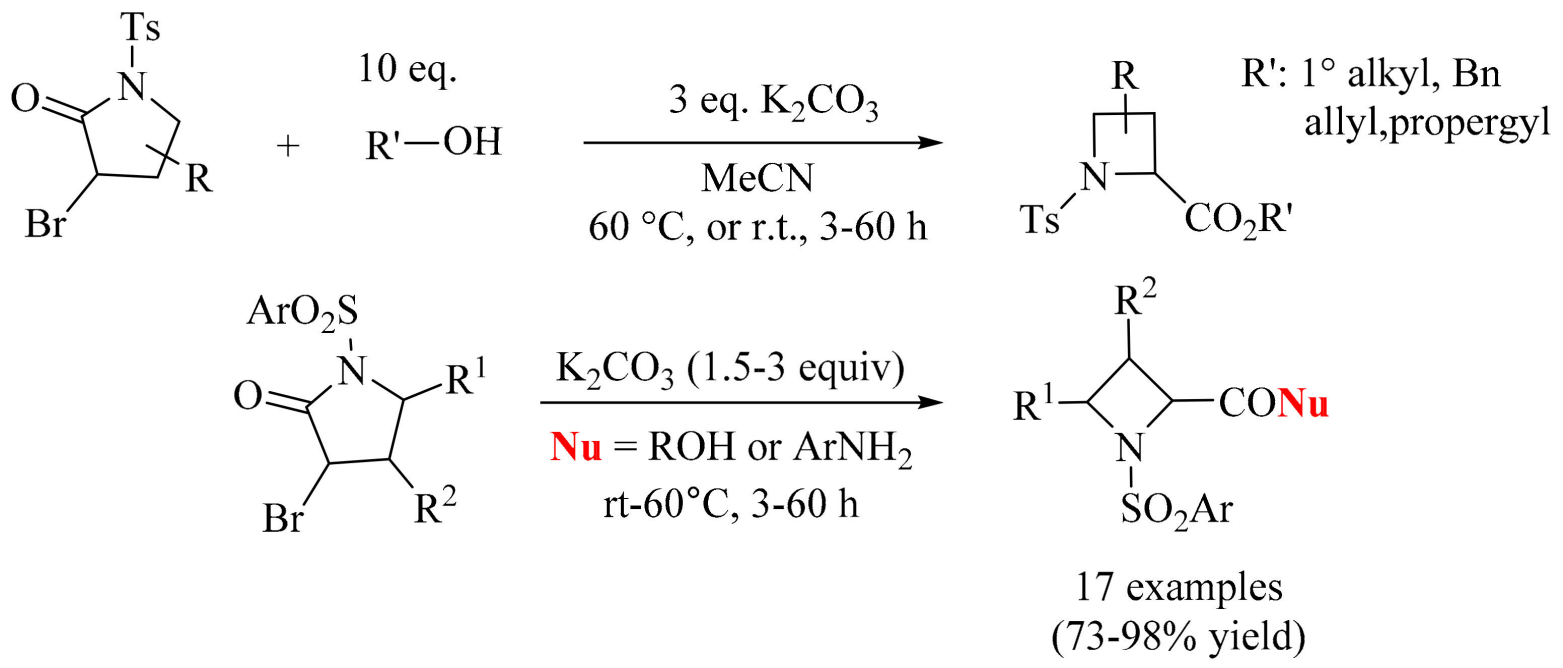
Synthesis of α-carbonylated *N*-sulfonylazetidines.

Kem et al. presented a robust one-pot procedure for the synthesis of α-carbonylated *N*-sulfonylazetidines. In the presence of potassium carbonate, the reaction involves the nucleophilic addition mediated ring contraction reactions of α-bromo *N*-sulfonylpyrrolidinones. They managed to synthesize variously substituted azitidine derivatives using nucleophiles like anilines, phenols and alcohols (Scheme 15) [31].

**Scheme 16:**
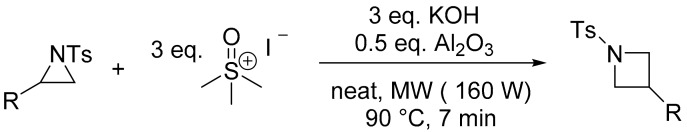
Synthesis of 1-arenesulfonylazetidines.

A general and efficient one-pot procedure has been presented by Malik et al. for the synthesis of 1-arenesulfonylazetidines. During this microwave-assisted reaction, the 1-arenesulfonylaziridines reacts with dimethylsulfoxonium methylide on a solid support of alumina to generate high yields of the product (Scheme 16) [32].

**Scheme 17:**
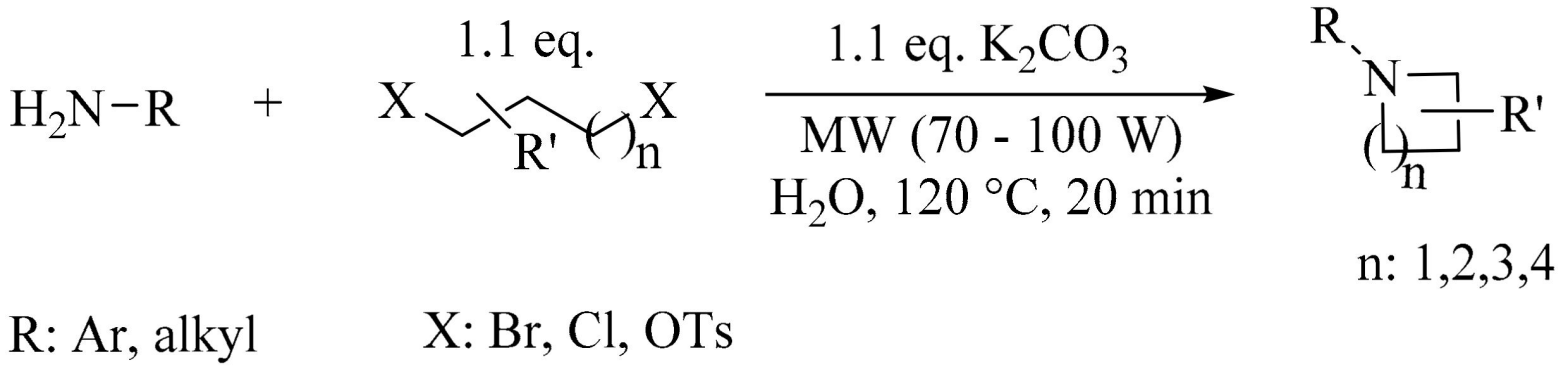
Microwave-assisted synthesis of cyclic amines.

An efficient, simple and one-pot cyclocondenation of primary amines and alkyl dihalides in an alkaline aqueous medium results in the formation of nitrogen-containing heterocycles. This microwave-assisted reaction is complete in just about 20 min (Scheme 17) [33].

**Scheme 18:**
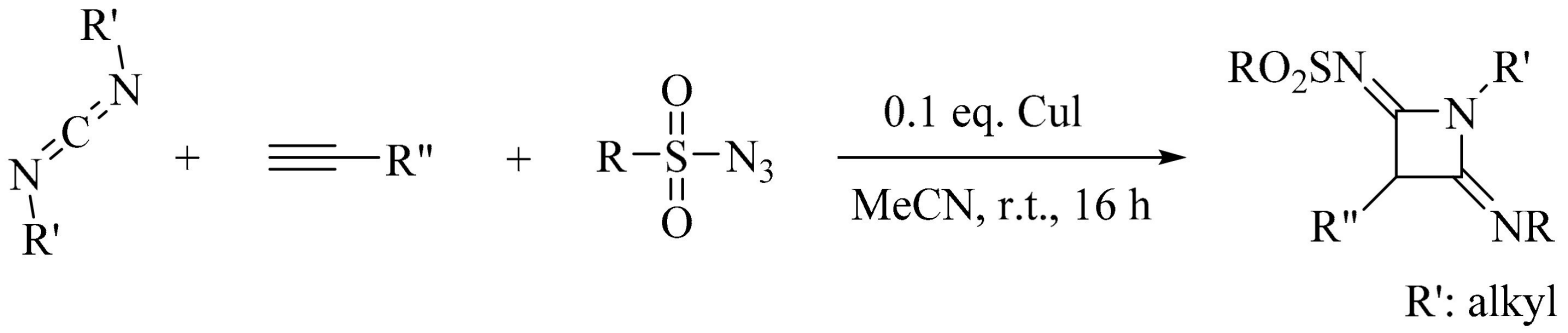
Synthesis of functionalized 2-(sulfonylimino)-4-(alkylimino)azetidines.

Under very mild conditions, the carbodiimides, sulfonyl azides and terminal alkynes react to generate high yields of functionalized 2-(sulfonylimino)-4-(alkylimino)azetidine derivatives. This multi-component reaction is catalyzed by Cu (Scheme 18) [34].

**Scheme 19:**
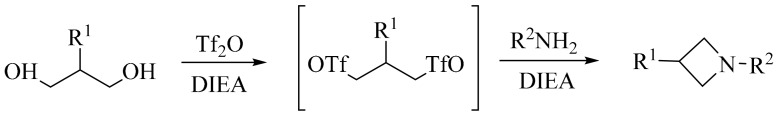
Synthesis of disubstituted azitidines.

Hillier and his colleague accomplished the synthesis of 1,3-disubstituted azetidines through an undemanding method. During this reaction, the bistriflate of a 2-substituted-1,3-propanediol helps to achieve alkylation of primary amines leading to the formation of disubstituted azitidines [35]. The scope of this procedure has been well established using a variety of amines and 2-substituted-1,3-propanediols (Scheme 19).

**Scheme 20:**
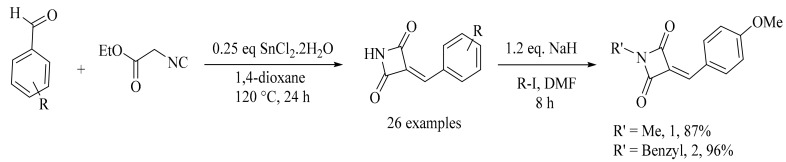
Synthesis of 3-substituted azetidine-2,4-diones.

A variety of 3-substituted azetidine-2,4-diones were synthesized when aromatic aldehydes underwent a tandem cyclization with ethyl cyanoacetate. This Sn(II)-catalyzed reaction proceeds through Knoevenagel condensation, hydration and the C-N cyclization to furnished good to excellent yields of the product (Scheme 20). This atomically economic reaction showed a broad substrate scope [36].

**Scheme 21:**
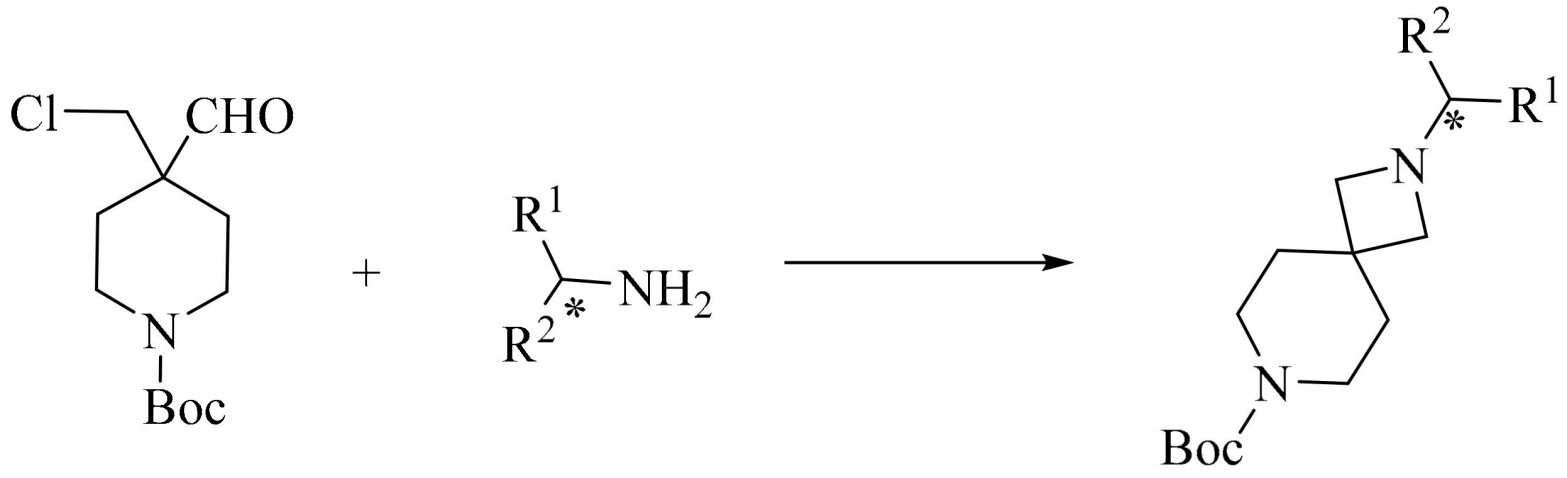
Synthesis of chiral azetidine-piperidines.

A facile, two-step and one-pot procedure provides an easy access to a range of chiral azetidine-piperidines by the reaction of chiral amines and piperidine chloroaldehyde [37]. Interestingly, the product retain the chirality of starting amine (Scheme 21).

**Scheme 22:**
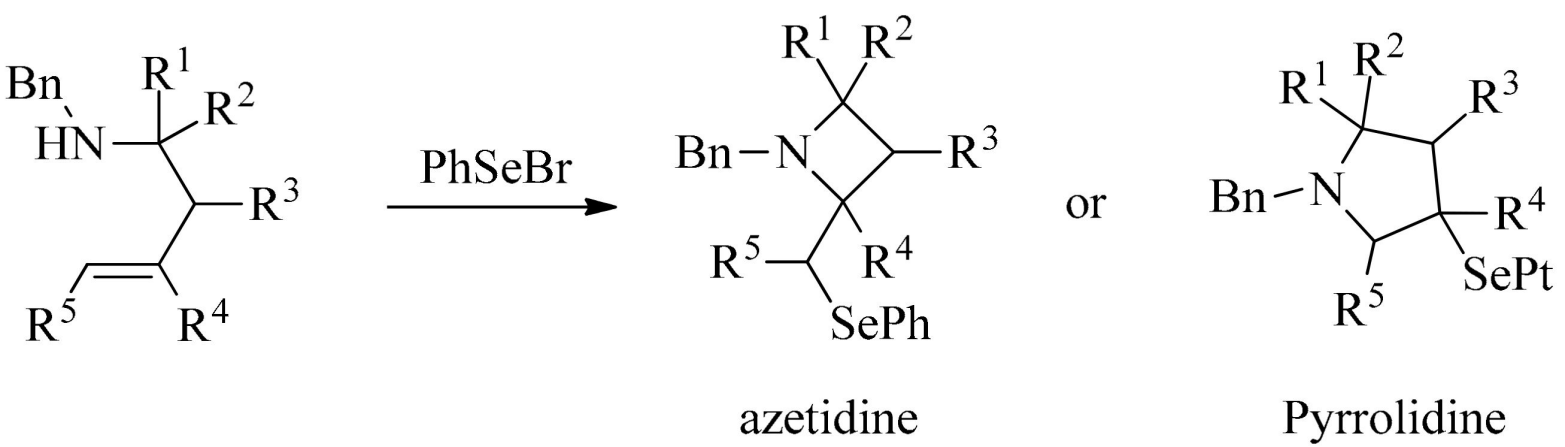
Regioselective procedure for the synthesis of azetidines or pyrrolidines.

Franck et al. described a straightforward and regioselective procedure for the synthesis of azetidines or pyrrolidines in good to excellent yields (Scheme 22). In this reaction the homoallylic amines undergo selenocyclization in respect of substitutions on double bond [38].

**Scheme 23:**
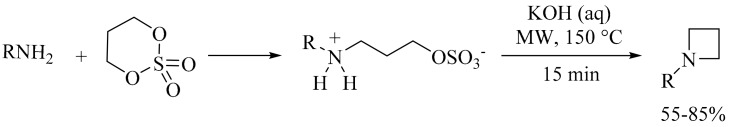
Microwave-assisted synthesis of azetidines in aqueous media.

Burkett and his colleagues presented a simple and facile microwave-assisted methodology for the accelerated synthesis of highly pure azetidines in aqueous media [39]. The reaction proceeds via the cyclization of 3-(ammonio)propyl sulfates derived from the cyclic sulfate of 1,3-propanediol and primary amines (Scheme 23).

**Scheme 24:**
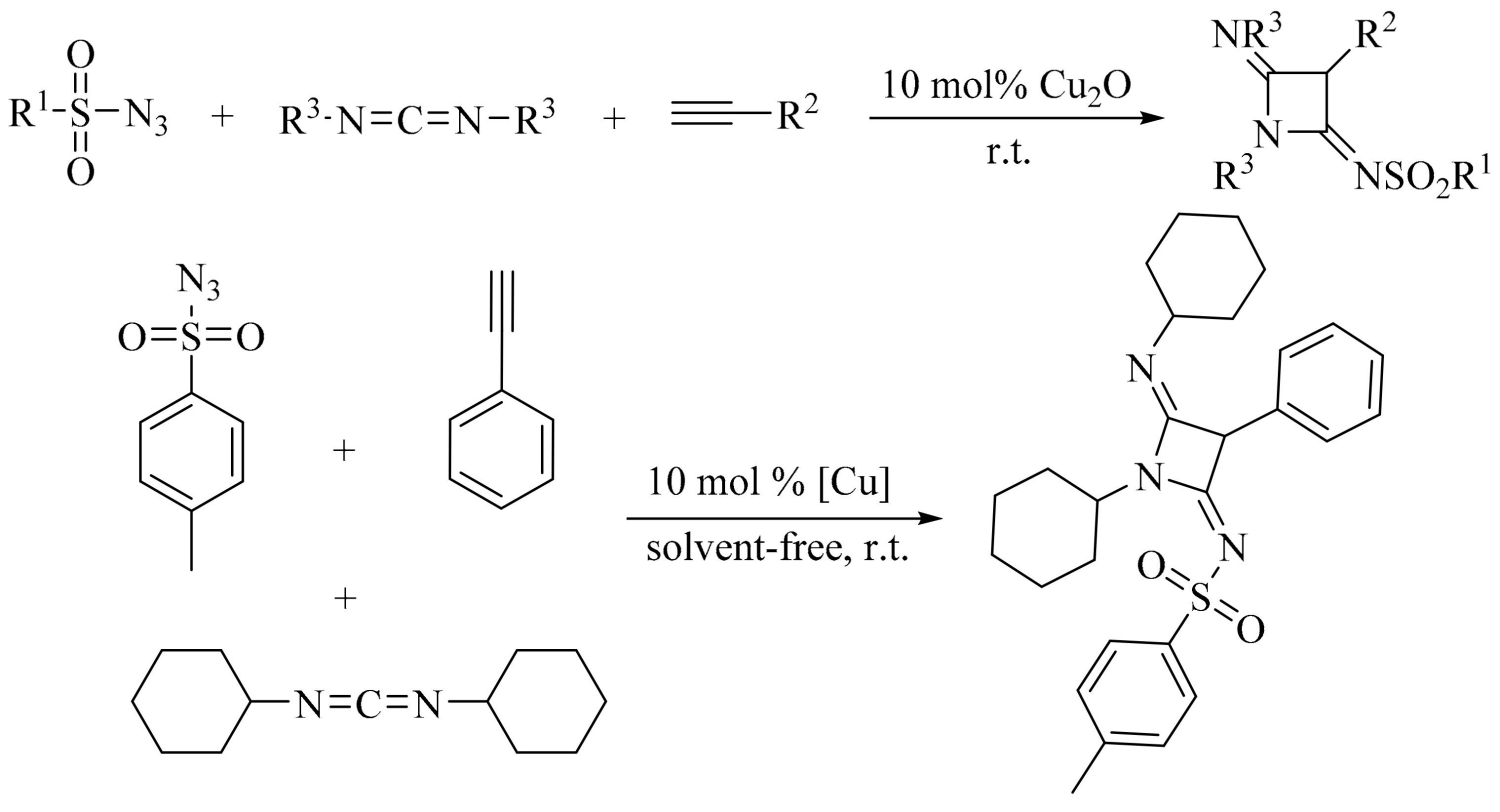
Synthesis of *N*-Sulfonylazetidin-2-imines.

Under solvent free conditions, the terminal alkynes, carbodiimides and sulfonyl azides reacts efficiently to generate *N*-Sulfonylazetidin-2-imines [40]. This one-pot three component reaction is catalyzed by an eco-friendly copper(I) oxide catalyst without the support of any ligand or base. The azetidin-2-ylidene derivatives have also been successfully prepared when 1,2-diamine were used as nucleophiles (Scheme 24).

## 4. Green Synthesis of Pyrrolidines

**Scheme 25:**
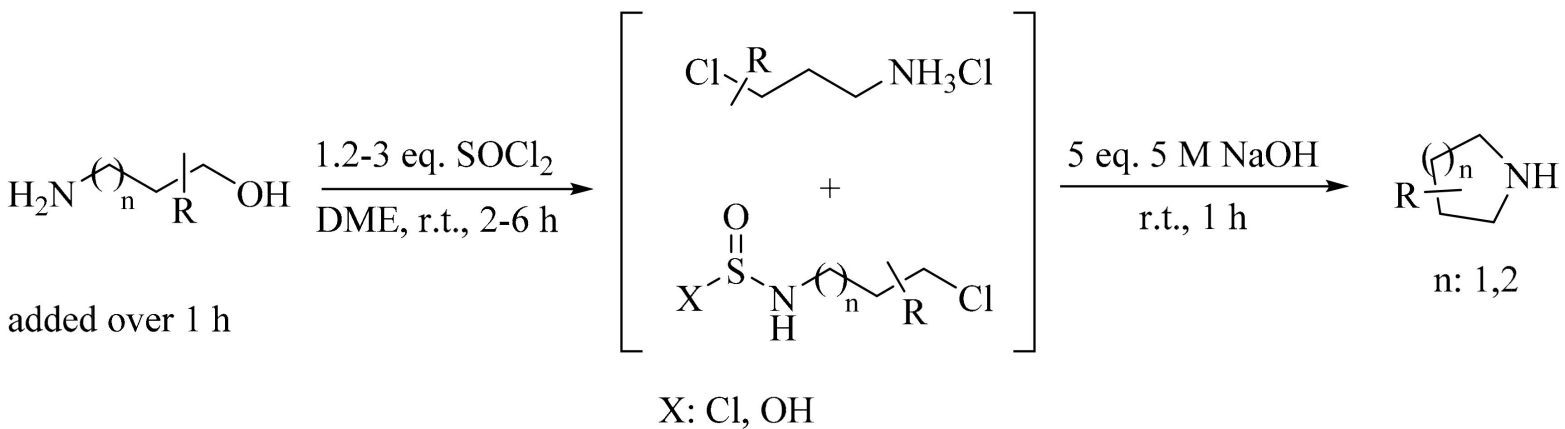
One-pot synthesis of cyclic amines.

Xu and his co-workers came up with a simple, efficient and one-pot procedure for the synthesis of cyclic amines. The reaction proceeds with the chlorination of amino alcohols and subsequently results in the formation of cyclic amines (Scheme 25). Here, the chlorination step obviates the use of common N-protection/O-activation/cyclization/deprotection steps required for such transformations. The scope of the reaction has been well-explored and the mechanistic aspects were studied in detail [41].

**Scheme 26:**
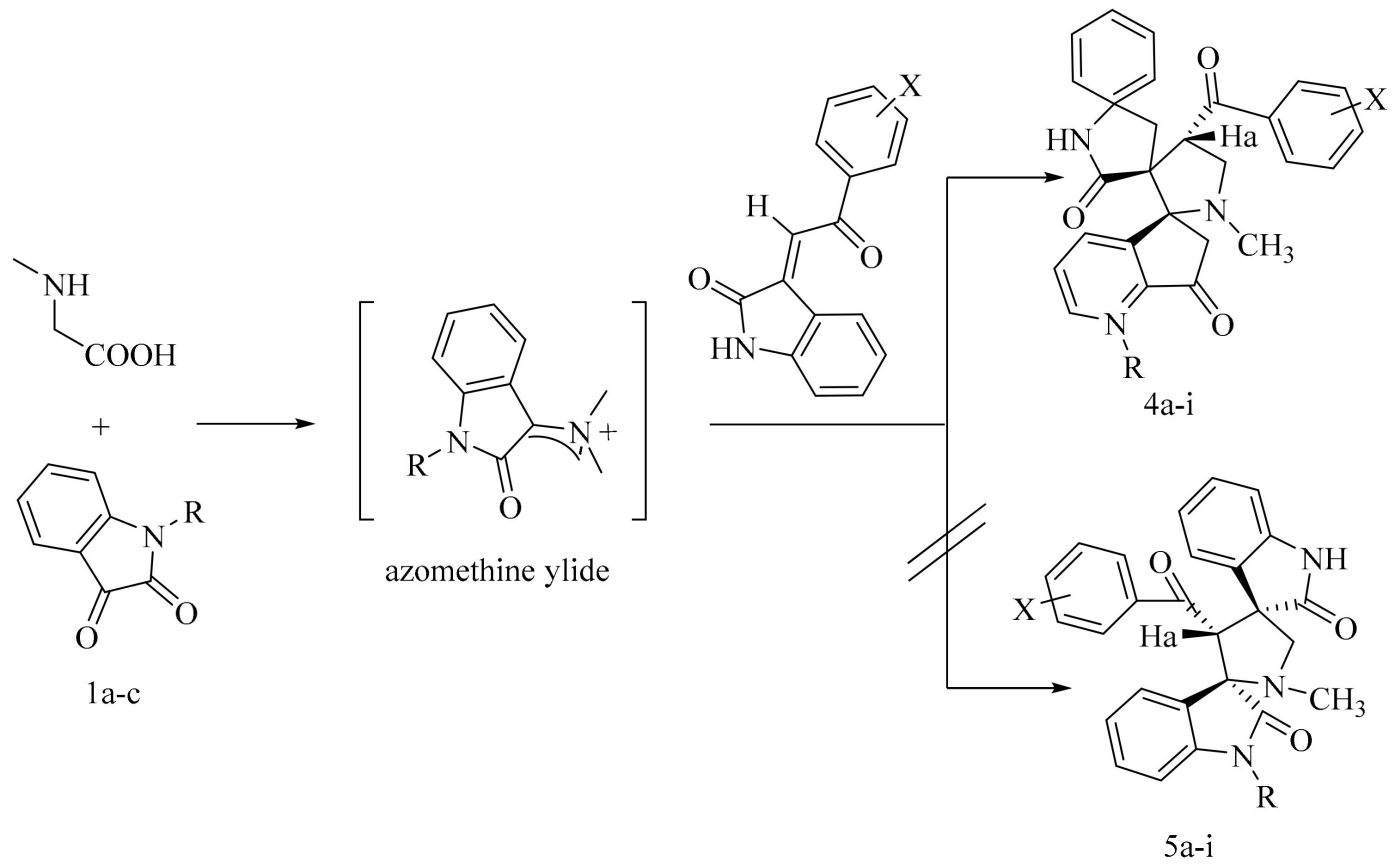
One-pot synthesis of dispiropyrrolidine-bisoxindole derivatives.

Jain and his colleagues reported a simple one-pot three-component reaction for an easy access to dispiropyrrolidine-bisoxindole derivatives. The reaction involves the cycloaddition trappings of azomethine ylides produced in situ through decarboxylative condensation of isatin with sarcosine (Scheme 26). This environmentally benign, operationally simple, highly regio- and stereoselective reaction completes in short time in ionic liquid solvent without the requirement of any catalyst [42].

**Scheme 27:**
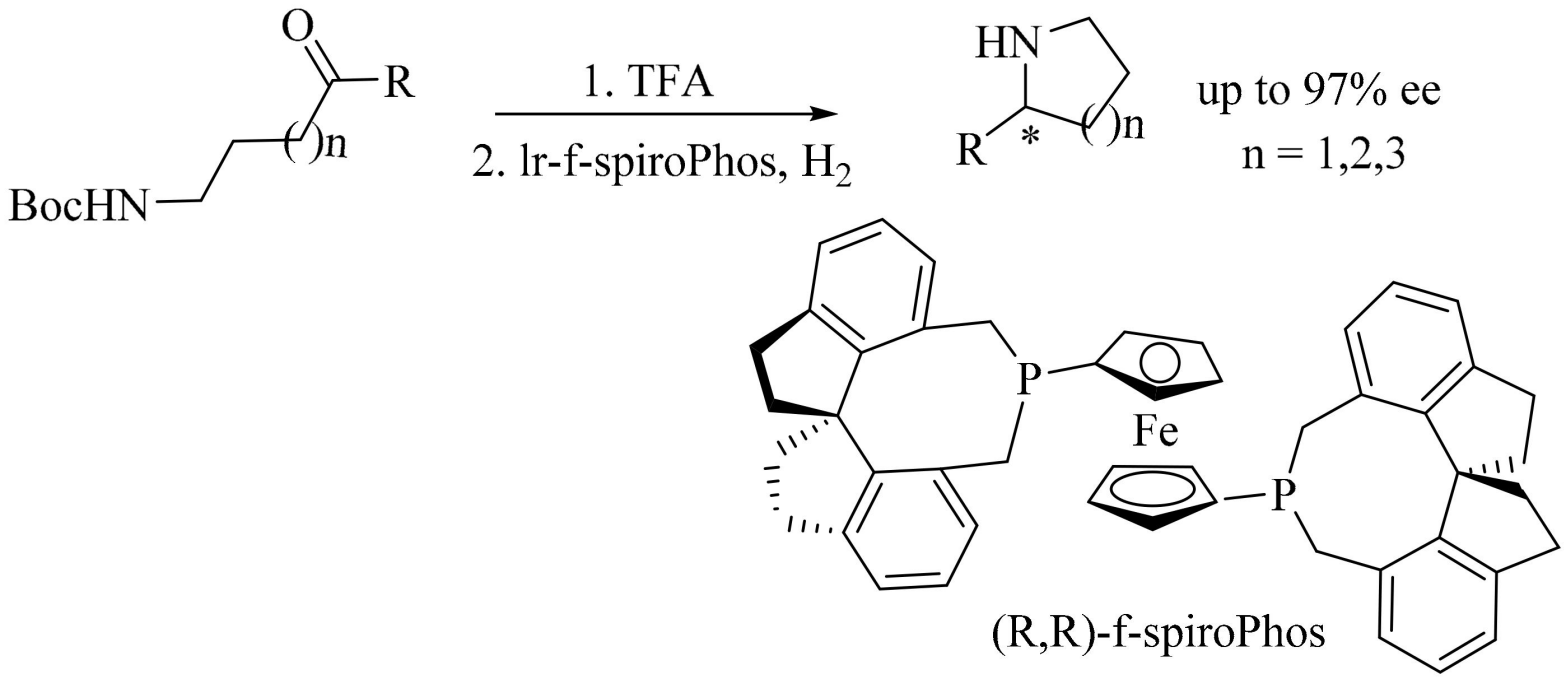
Synthesis of cyclic amines from *N*-Boc protected amino ketones.

Zhang et al. presented a one-pot highly enantioselective methodology for the synthesis of cyclic amines including pyrrolidine, piperidine and azocane via intramolecular reductive amination of *N*-Boc-protected aminoketones (Scheme 27) [43].

**Scheme 28:**
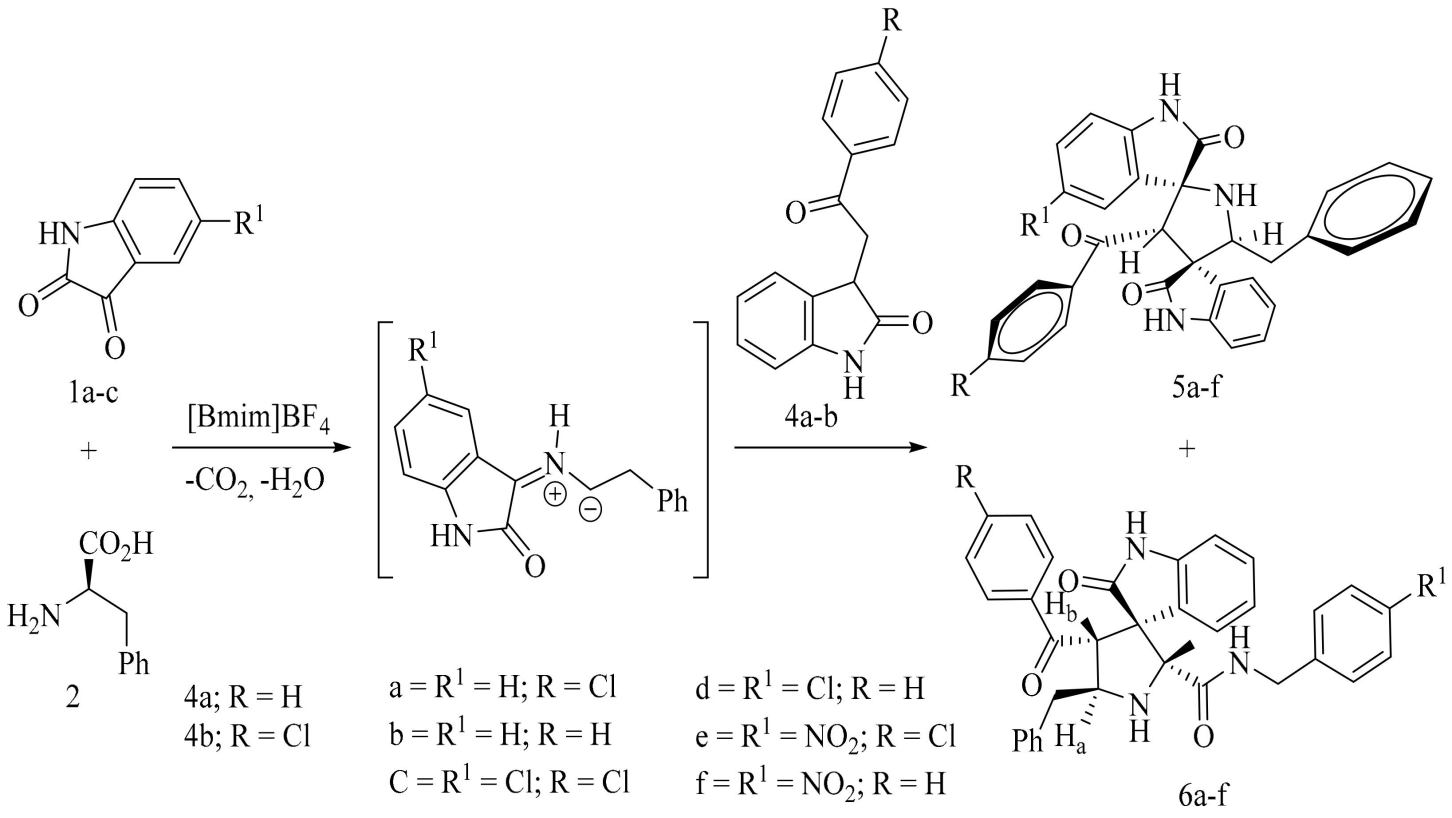
One-pot synthesis of dispirooxindolo-pyrrolidines.

Abdulrahman et al. also reported the synthesis of dispirooxindolo-pyrrolidines in good yields with high regioselectivities. The one-pot three component 1,3-dipolar cycloaddition methodology involves the reaction between in situ generated azomethine ylides from L-phenylalanine with substituted isatin with unusual (*E*)-2-oxoindolino-3-ylidene acetophenone dipolarophiles (Scheme 28). The reaction is run in recyclable ionic liquids [44].

**Scheme 29:**
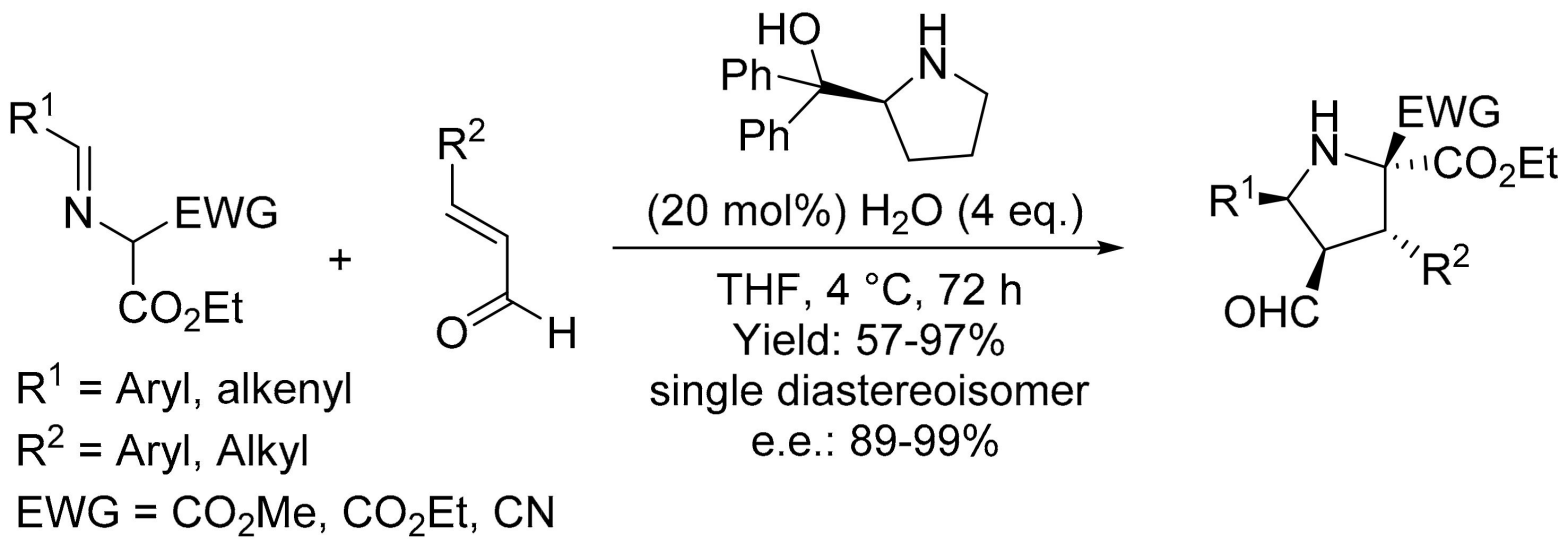
Synthesis of substituted-pyrrolidines.

Ugarriza et al. presented an excellent methodology for the synthesis of C3-unsubstituted pyrrolidines with about 80% enantiomeric control and complete diastereomeric control. The in situ formed acyclic azomethine ylides react with acrolein through [3 + 2]-cycloadditions to furnish high yields of the product (Scheme 29). The reaction was catalyzed by easily available chiral amines like L-proline [45].

**Scheme 30:**
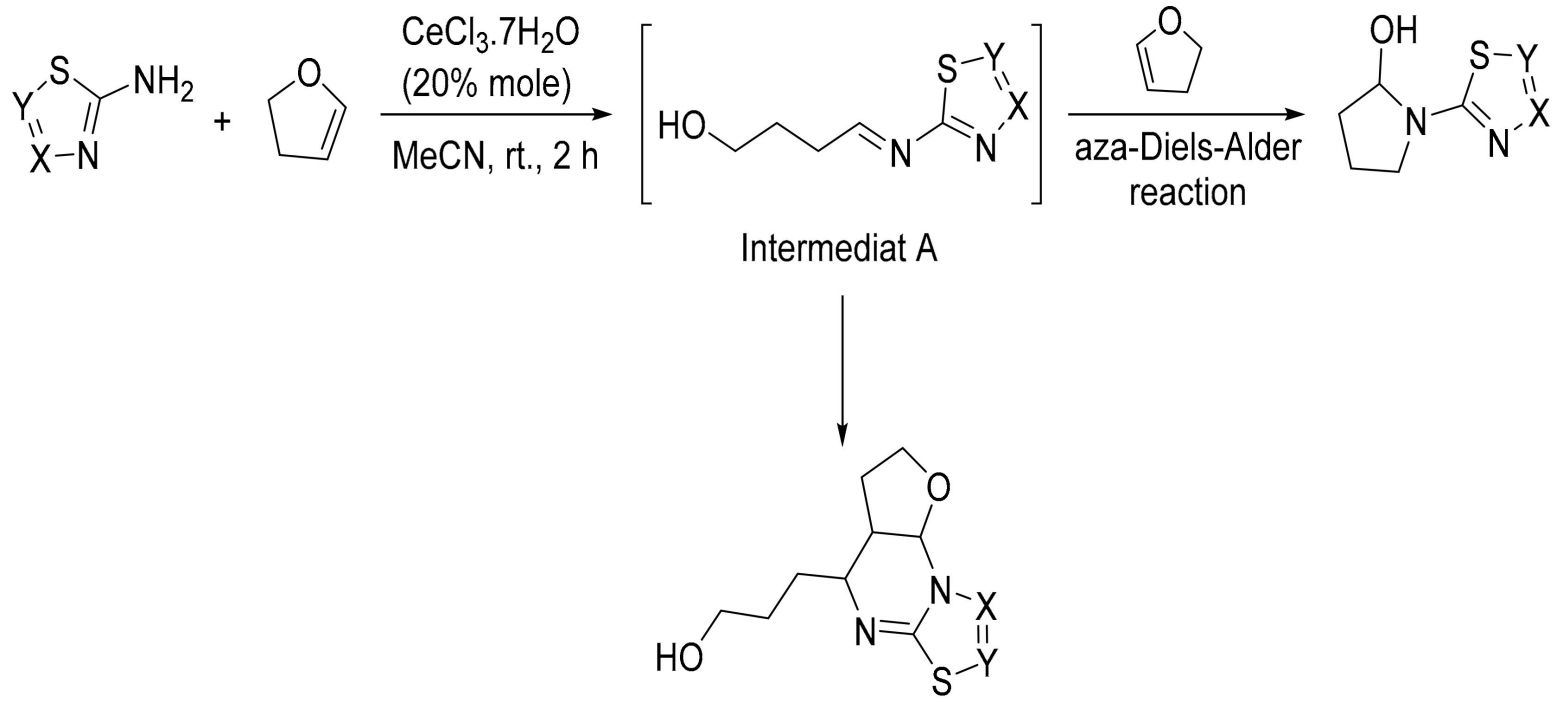
One-pot synthesis of 2-hydroxypyrrolidines.

Varma and his research colleagues developed a one-pot methodology for a convenient synthesis of 2-hydroxy pyrrolidines under mild reaction conditions [46]. In the presence of CeCl_3_·7H_2_O as catalyst, different 2-aminothiazoles or thiadiazoles and 2,3-dihydrofuran reacts to furnish 2-hydroxy- pyrrolidines (Scheme 30).

**Scheme 31:**
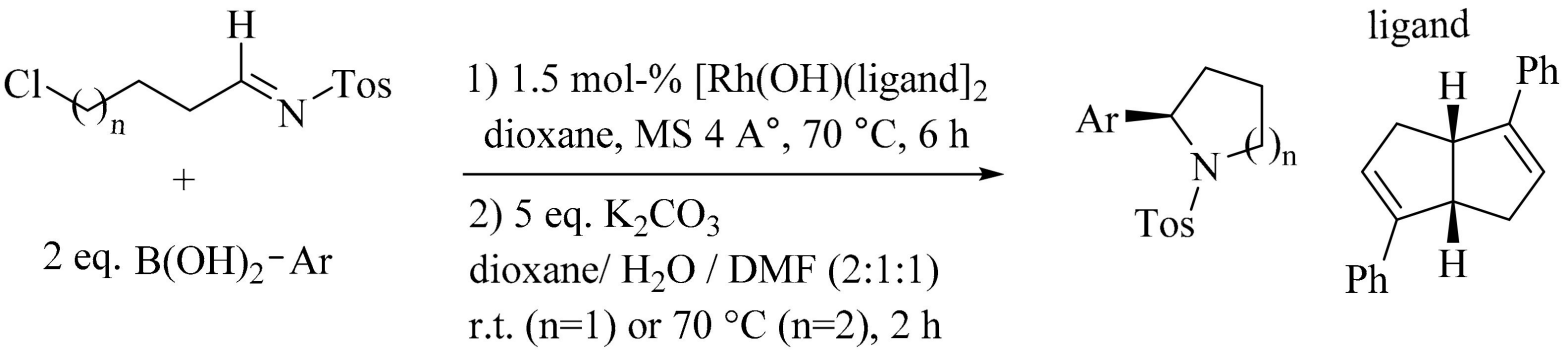
Synthesis of chiral 2-aryl pyrrolidines and piperidines.

Cui and his research team reported a one-pot methodology for enantioselective synthesis of chiral 2-aryl pyrrolidines and piperidines. Under neutral reaction conditions, rhodium hydroxide complex in the presence of chiral bicyclo[3.3.0]octadiene ligands catalyzes arylation of aliphatic *N*-tosylaldimines (Scheme 31). This high yielding method has been adopted for enantioselective synthesis of chiral 2-aryl pyrrolidines and piperidines [47].

**Scheme 32:**
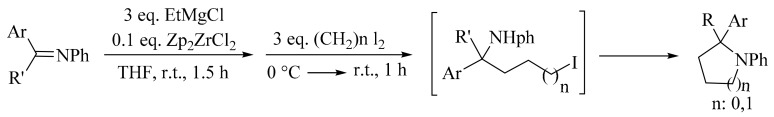
Synthesis of bifunctional cyclic amines.

In 2002, Gandon and Szymoniak reported an excellent procedure to access bifunctional nitrogen-heterocycles such as 1-azaspirocyclic γ-lactams, azetidines and pyrrolidines [48]. Imines react with EtMgCl under Zr-catalyzed reaction conditions to furnish C,N-dimagnesiated compounds which could be trapped with electrophiles leading to aforementioned N-heterocycles (Scheme 32).

**Scheme 33:**
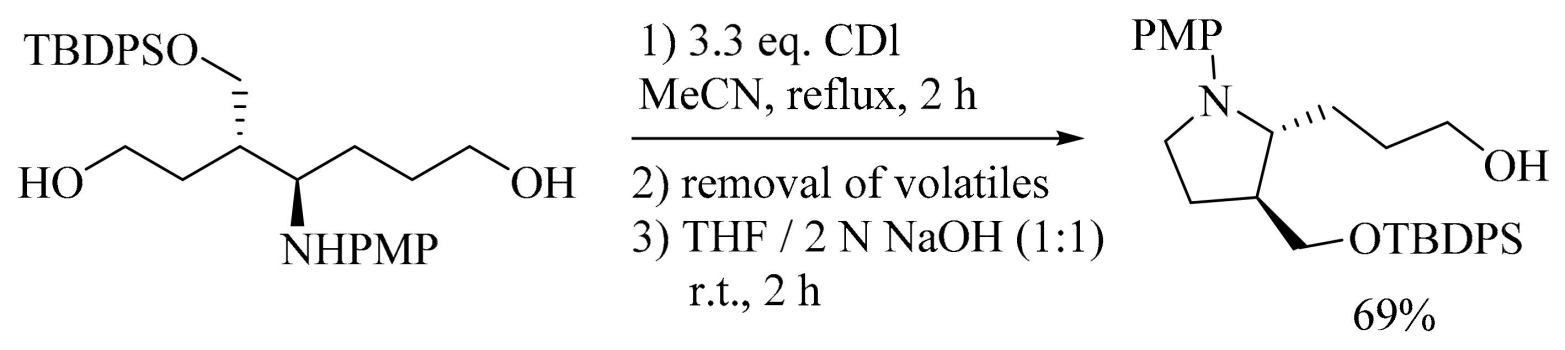
Synthesis of functionalized *p*-methoxyphenyl-protected 4-, 5- and 6-membered *N*-heterocycles.

In 2006, de Figueiredo and his colleagues described an efficient method for the synthesis of functionalized *p*-methoxyphenyl-protected 4-, 5- and 6-membered *N*-heterocycles (Scheme 33). This strategy involves the activation of hydroxyl group of amino alcohol without the use of toxic reagents. The scope of the reaction has been well-established by its ability to tolerate various functional groups [49].

**Scheme 34:**
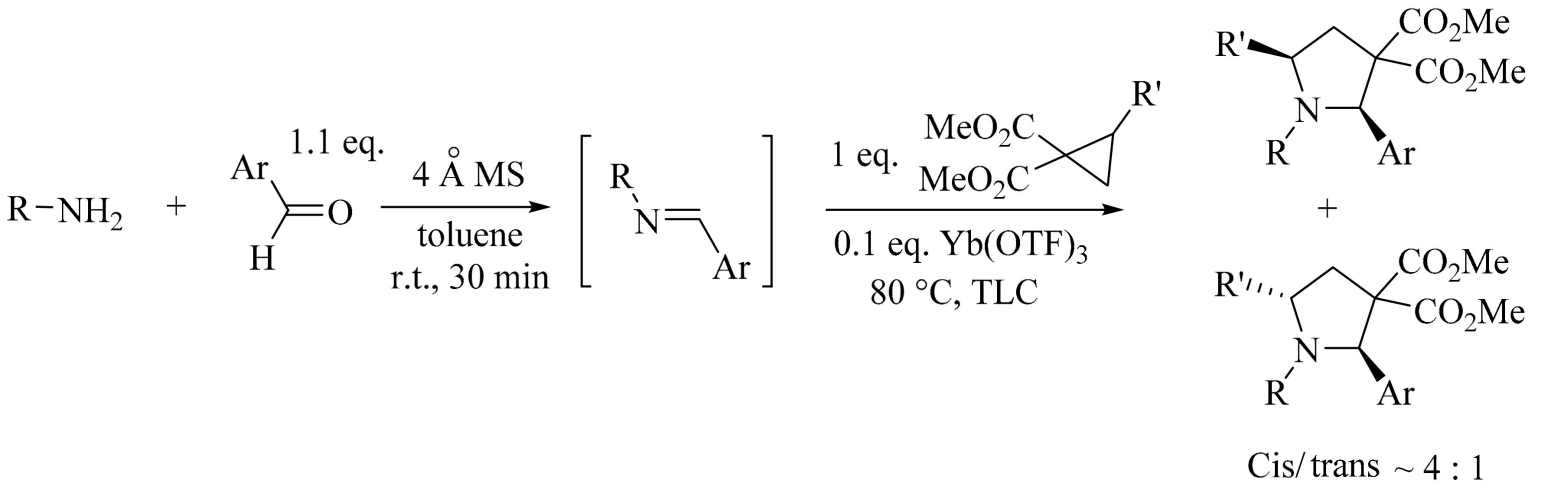
Synthesis of substituted pyrrolidines.

Carson and his colleagues presented an excellent one-pot three-component procedure for the synthesis of substituted pyrrolidines [50]. During this reaction, the condensation of anilines or primary amines with aldehydes generates aldimines in situ which subsequently react with 1,1-cyclopropandiesters to afford pyrrolidines (Scheme 34). This high yielding-reaction is catalyzed by Yb(OTf)_3_.

**Scheme 35:**
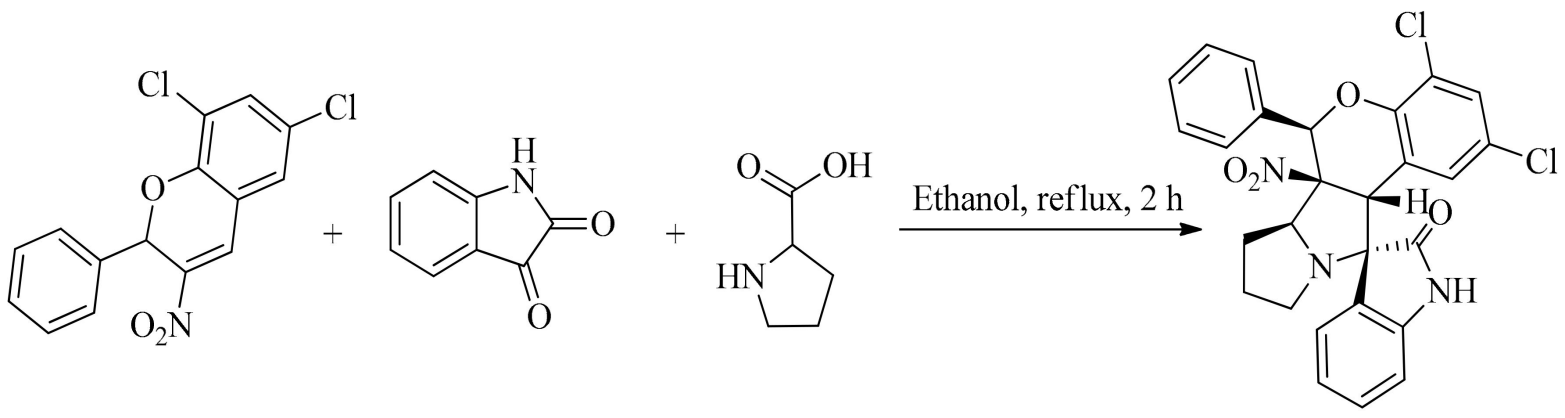
Synthesis of spirooxidindole-pyrrolidine/piperidine fused nitrochromanes.

Nayak and her research group described an operationally simple, eco-friendly and novel one-pot three component reaction for the synthesis of spirooxidindole-pyrrolidine/piperidine fused nitrochromanes in good to excellent yields (Scheme 35). The less demanding reaction procedure involves the cycloaddition of isatin-based azomethine ylide and substituted nitrochromenes in nontoxic solvent in shorter time [51].

**Scheme 36:**
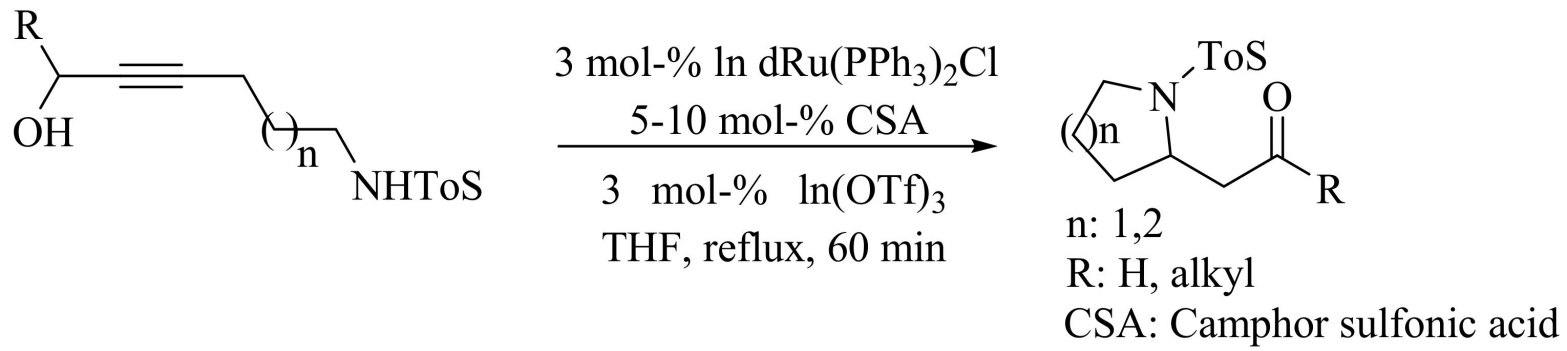
Domino synthesis of cyclic amines.

Trost and his colleagues described that aminopropargyl alcohols undergo domino redox isomerization/cyclization to furnish nitrogen heterocycles [52]. In this ruthenium-catalyzed, atom-economical single step reaction a variety of functional groups were tolerated, establishing the broad scope of reaction (Scheme 36).

**Scheme 37:**
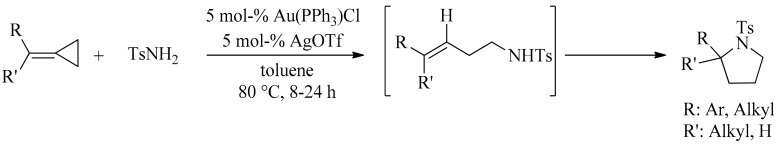
Gold(I)-catalyzed synthesis of pyrrolidine derivatives.

Methylenecyclopropanes react with sulfonamides through a gold(I)-catalyzed domino ring-opening ring-closing hydroamination process to afford pyrrolidine derivatives in excellent yield (Scheme 37) [53].

**Scheme 38:**
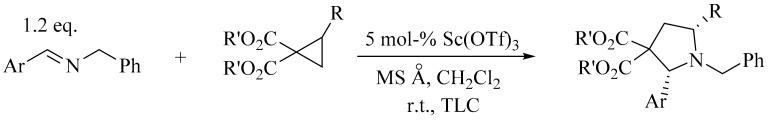
Synthesis of multisubstituted pyrrolidines by tandom reaction.

Multisubstituted pyrrolidines were made accessible with high diastereoselectivity by the tandem ring-opening-cyclization reaction of imines with cyclopropanes (Scheme 38). Kang and his research team used 5 mol% of scandium triflate as catalyst for the reaction [54].

**Scheme 39:**
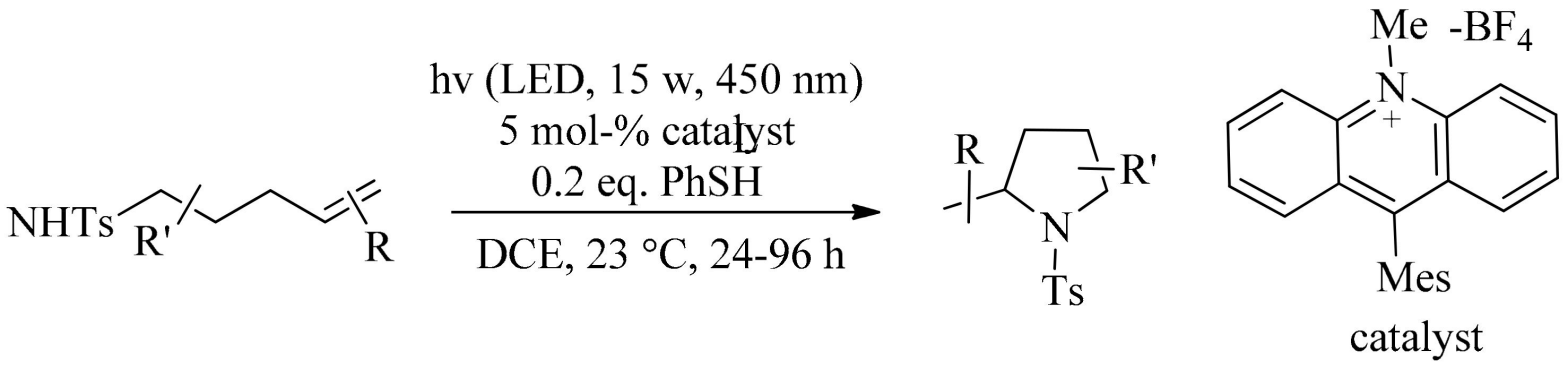
Metal-free synthesis of nitrogen-containing heterocyles.

Nguyen and Nicewicz presented a metal-free procedure for the synthesis of nitrogen-containing heterocyles with complete regiocontrol. The reaction involves a direct anti-Markonikov hydroamination of unsaturated amines (Scheme 39). In the presence of 9-mesityl-10-methylacridinium tetrafluoroborate as catalyst, the substrate amines are irradiated with visible light and thiophenol manages to donate H-atom [55].

**Scheme 40:**
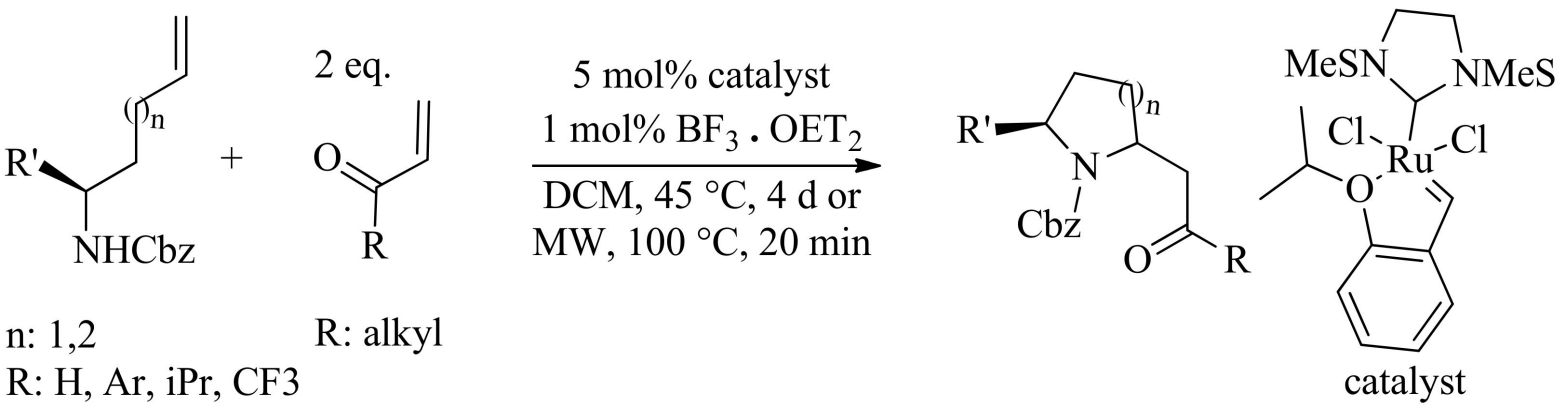
Microwave assisted synthesis of pyrrolidines.

Enones and unsaturated carbamates undergo a tandem cross-metathesis intramolecular aza-Michael reaction to generate β-amino carbonyl compounds (Scheme 40). The reaction is efficiently promoted by a combination of BF_3_·OEt_2_ and Hoveyda-Grubbs catalyst. In this reaction, a dramatic acceleration is observed under microwave irradiation with inversion of stereoselectivity in the addition step [56].

**Scheme 41:**
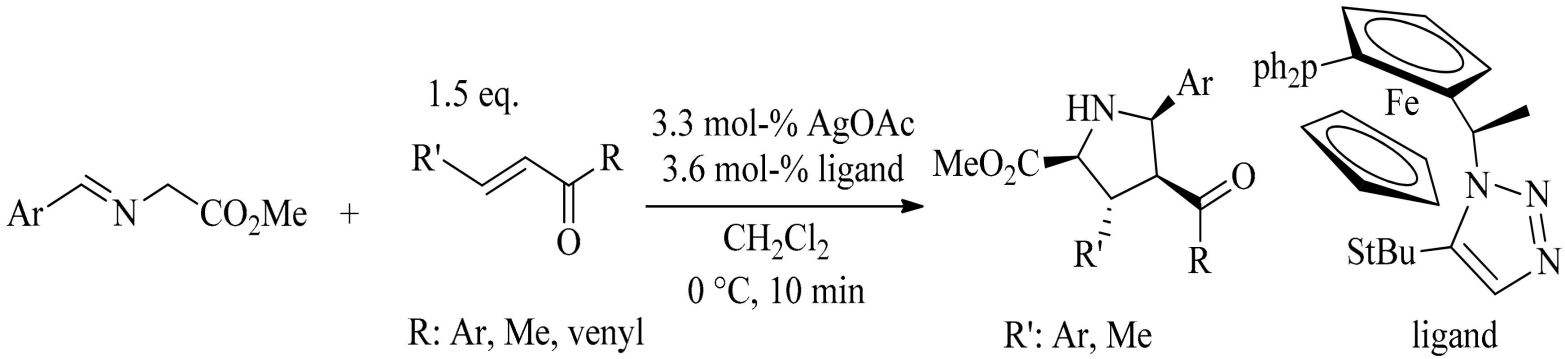
Synthesis of highly functionalized *endo*-4-acyl pyrrolidines.

A highly *endo*-selective asymmetric 1,3-dipolar cycloaddition reaction of methyl *N*-benzylideneglycinate as source of azomethine ylides with (*E*)-acyclic α-enones is catalyzed by a silver(I)/ThioClickFerrophos complex to give highly functionalized *endo*-4-acyl pyrrolidines in good yields with high enantioselectivities (Scheme 41) [57].

## 5. Green Synthesis of Piperidines

**Scheme 42:**
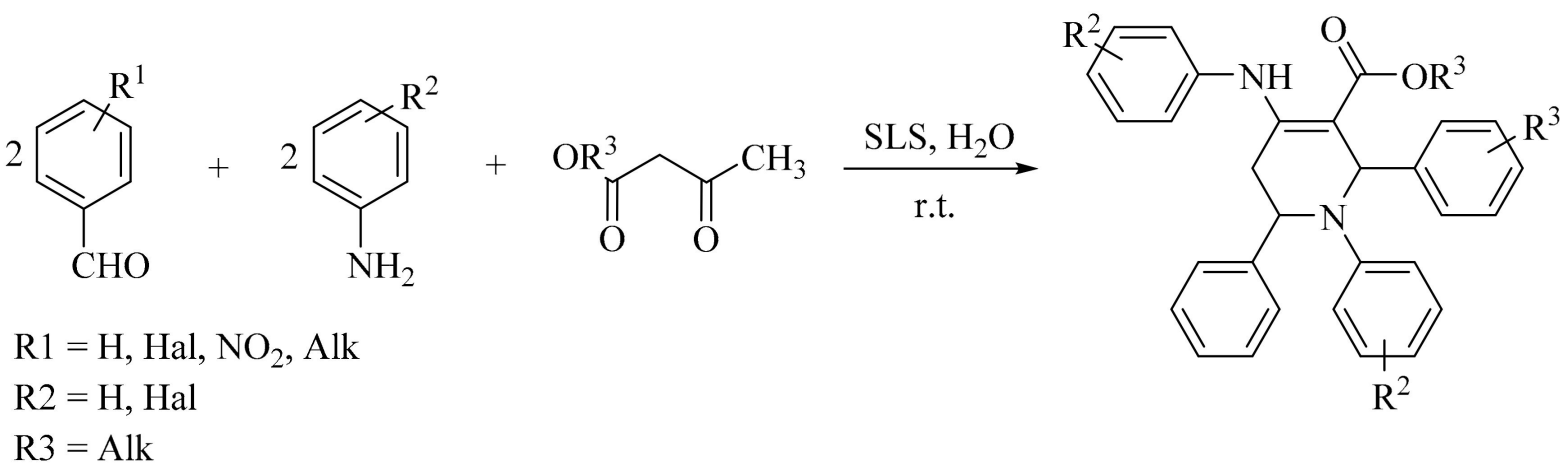
One-pot multicomponent synthesis of highly functionalized piperidines.

Bansal et al. developed one-pot multicomponent reaction to access highly functionalized piperidines in moderate to good yields (Scheme 42). The reaction involves the condensation of amines, aldehydes and β-ketoesters catalyzed by a recyclable, nontoxic, and eco-friendly sodium lauryl sulfate. As an advantage, the reaction completes in water at room temperature and requires simple workup to furnish highly substituted piperidines [58].

**Scheme 43:**
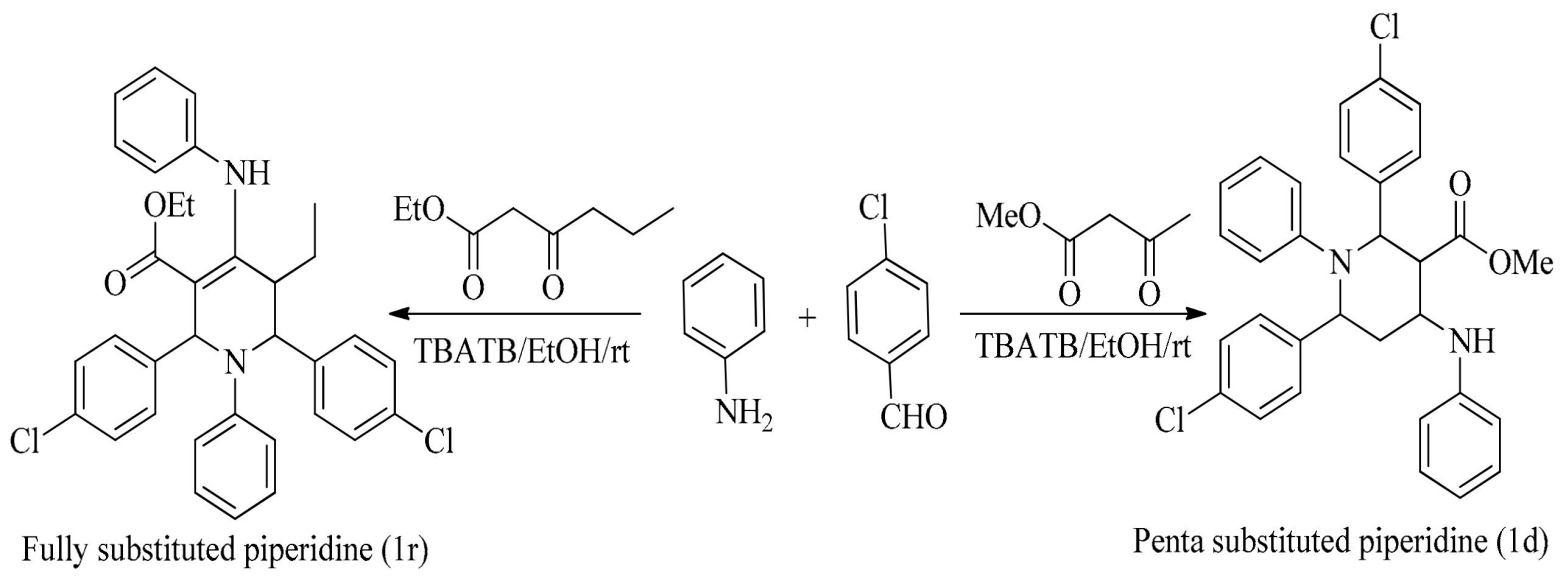
Synthesis of highly functionalized and fully substituted piperidines.

The abovementioned reaction (Scheme 42) had also been studied by Khan et al. for the highly functionalized piperidine as well for fully substituted piperidines (Scheme 43). The described one-pot multicomponent procedure offered a green approach due to atom economy, mild conditions and good yields [59].

**Scheme 44:**
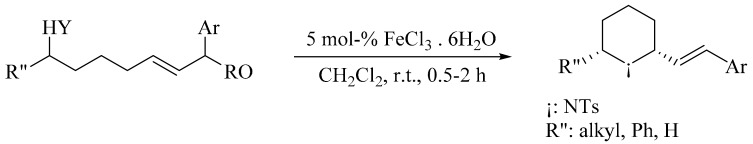
Synthesis of substituted *cis*-2,6-piperidines.

In 2010, Guérinot et al. represented an eco-friendly methodology for the synthesis *cis*-2,6-tetrahydropyrans and substituted *cis*-2,6-piperidines in good to excellent yields (Scheme 44). In this highly diastereoselective iron catalyzed reaction, the most stable *cis*-isomers are easy to isolate and purify [60]. 

**Scheme 45:**
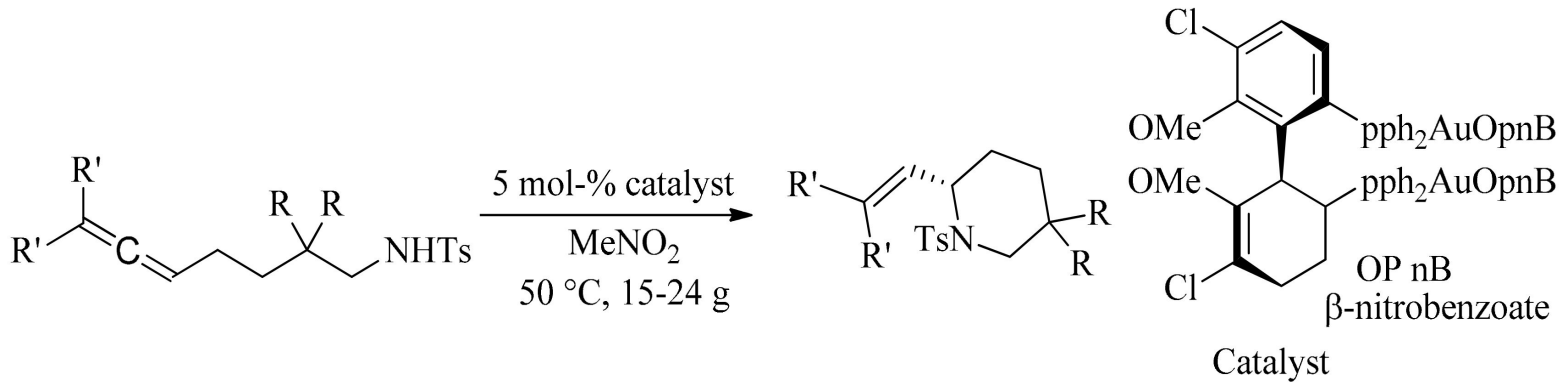
Synthesis of vinyl piperidines.

LaLonde and his co-workers described phosphinegold(I)-bis-*p*-nitrobenzoate complex catalyzed intramolecular hydroamination of alkenes to access vinyl pyrrolidines and piperidines (Scheme 45). This atom-economical and enantioselective reaction results in high ee [61].

**Scheme 46:**
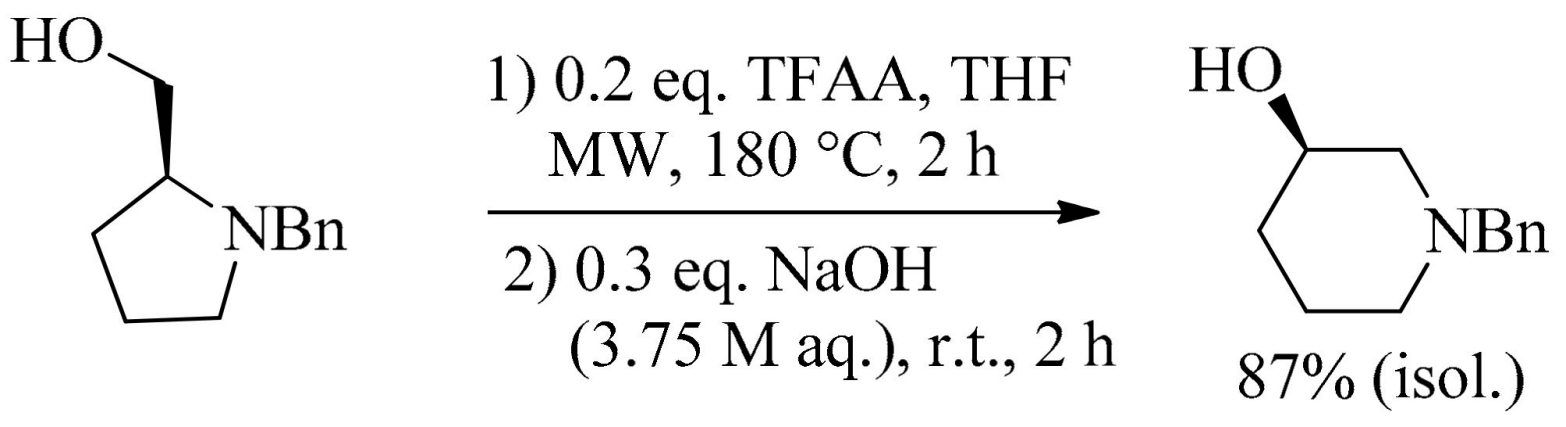
Microwave-assisted enantioselective synthesis of piperidines.

Métro and his research colleagues presented a high-yielding microwave-assisted reaction for the enantioselective synthesis of piperidines (Scheme 46) [62].

**Scheme 47:**
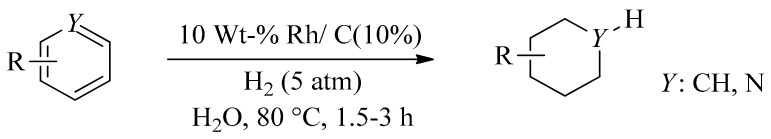
Synthesis of piperidines in aquous media.

Maegawa and his fellows presented an excellent methodology for mild and complete hydrogenation of various carbon and heteroatomic compounds including furans, biphenyls, pyridines, alkylbenzenes, etc. [63]. The same reaction has been adopted for the synthesis of piperidines in good to excellent yield in aqueous media (Scheme 47).

## 6. Green Synthesis of Azepanes

**Scheme 48:**
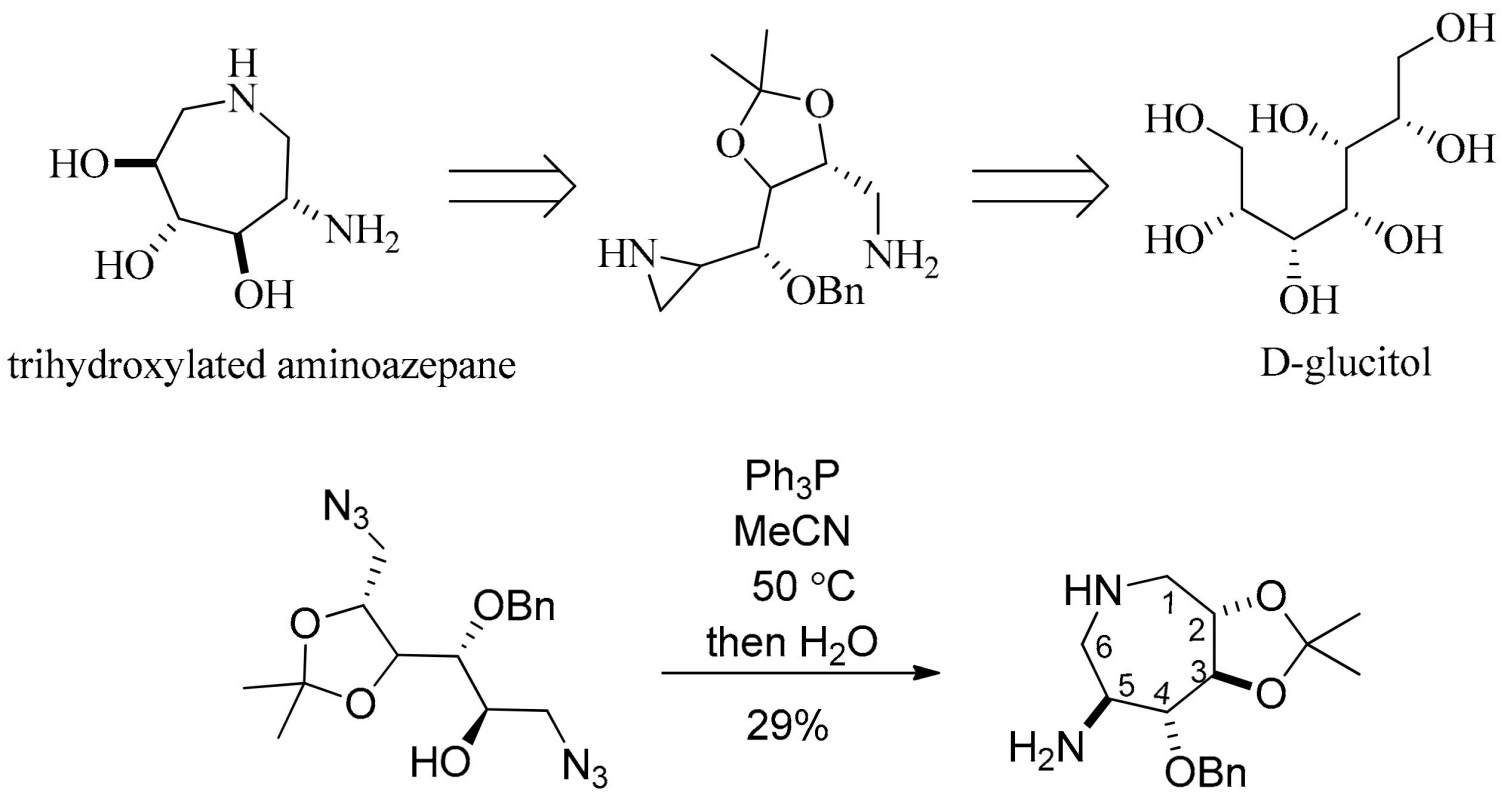
Synthesis of polyhydroxylated azopanes.

Valle and his colleague reported a one-pot synthesis of polyhydroxylated aminoazepanes using Ph_3_P in aqueous solvent. In the reaction, the D-glucitol was initially converted into 1,6-diazido derivatives which subsequently got converted into the product (Scheme 48) [64].

**Scheme 49:**
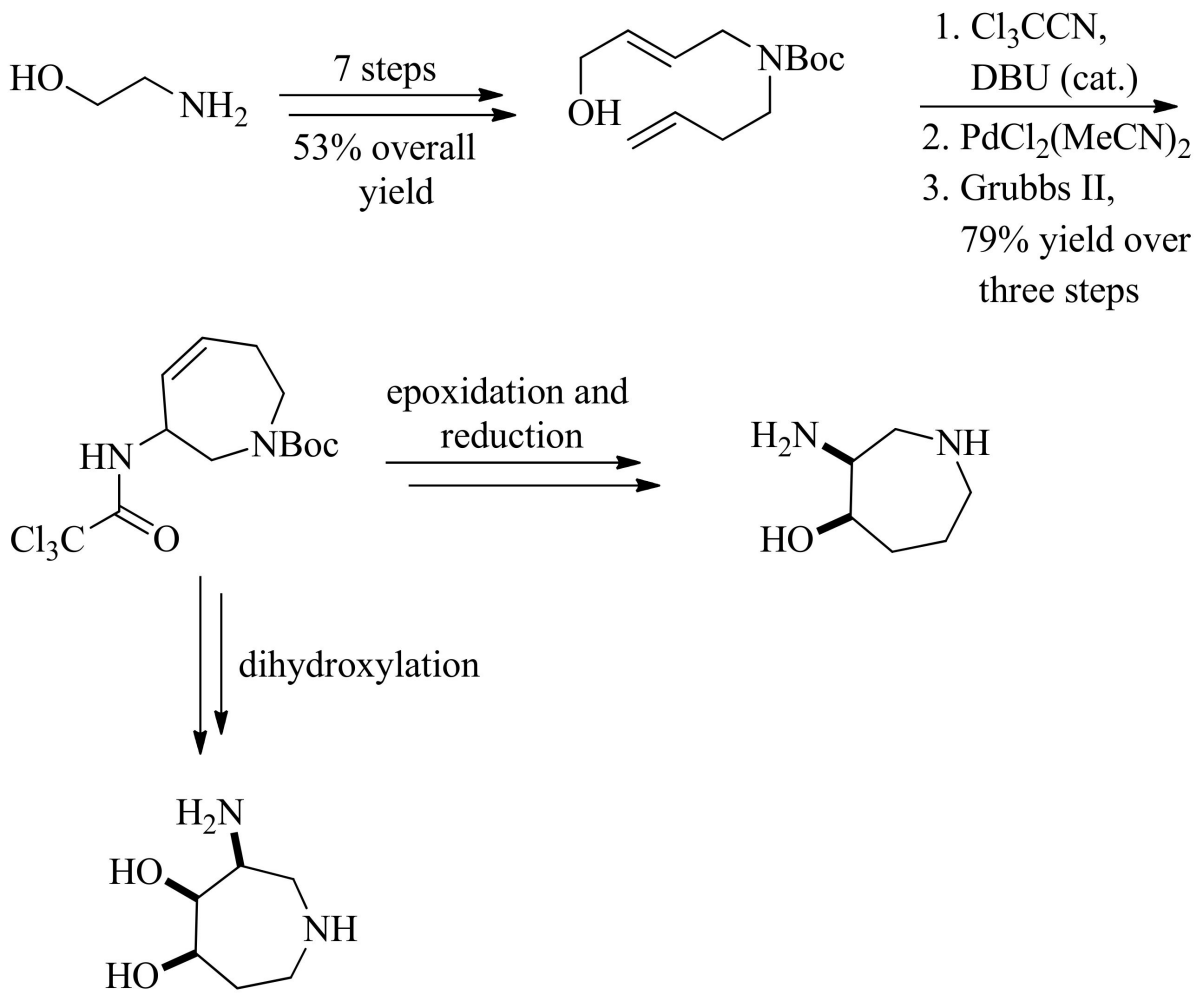
Synthesis of hydroxylated 3-aminoazepanes.

Calder et al. presented a one-pot multi-reaction strategy for the synthesis of hydroxylated 3-aminoazepanes (Scheme 49) [65].

**Scheme 50:**
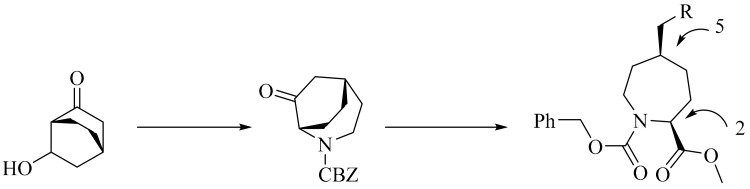
Synthesis of (2*S*,5*S*)-5-substituted-azepane-2-carboxylate derivatives.

Wishka and his colleagues presented a successful method for the synthesis of (2*S*,5*S*)-5-substituted-azepane-2-carboxylate derivatives starting from known hydroxyl ketone (Scheme 50) [66].

**Scheme 51:**
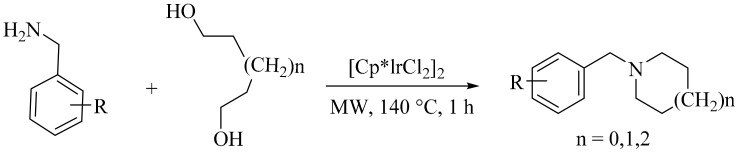
Microwave assisted iridium catalyzed synthesis of cyclic amines.

Zhang and his co-workers described the alkylation of amines with alcohol for the synthesis of cyclic amines (Scheme 51). This microwave assisted iridium catalyzed reaction recognized as a green reaction because of its features like atom-economy, solvent-free and base-free conditions [67].

Further, the green and facile methods including one-pot, domino and microwave assisted reactions for the synthesis of azopane has already been described in Scheme 28, Scheme 30, Scheme 34, Scheme 39 and Scheme 43.

## 7. Green Synthesis of Azocanes

**Scheme 52:**
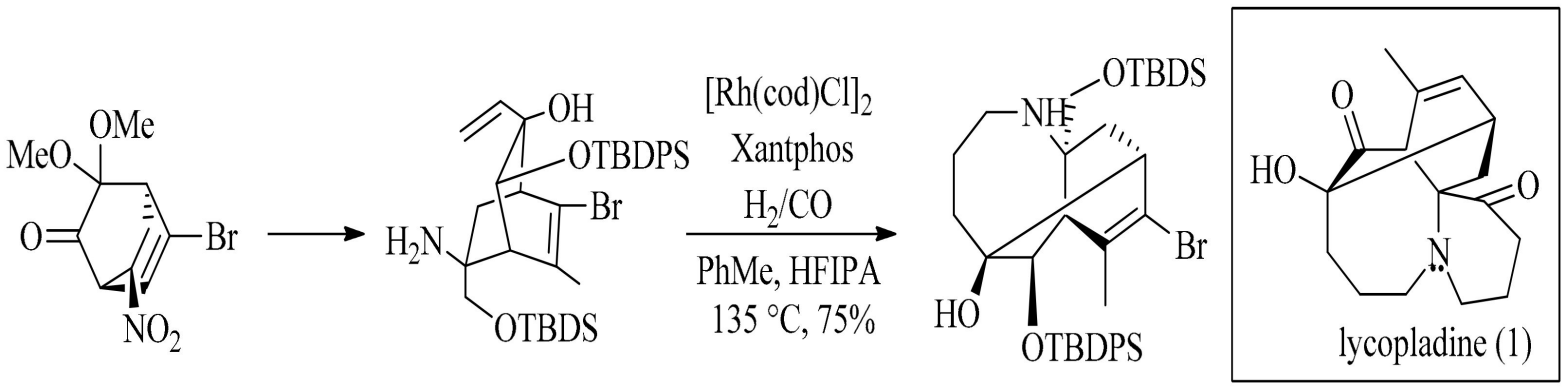
Atom economical synthesis of azocane.

Sacher and his fellow developed following atom economical methodology for the construction of the azocane ring (Scheme 52) [68].

The green synthesis of azocanes has also been described in Scheme 27 and Scheme 31.

## 8. Conclusions

In conclusion, the presence of saturated cyclic amines in a number of naturally occurring bioactive molecules and in synthetic drugs has indicated their various potential roles in the past few decades. Further, vast arrays of applications in various scientific disciplines have triggered the development of new methods for their facile synthesis. This manuscript has highlighted some eco-friendly and facile synthetic routes for the synthesis of unsubstituted, fully substituted and functionalized aziridines, azetidines, pyrrolidines, piperidines, azepanes and azocanes.
